# Supplementary material for: Maspin is a PTEN-Upregulated and p53-Upregulated Tumor Suppressor Gene and Acts as an HDAC1 Inhibitor in Human Bladder Cancer
Source: Cancers (Basel). 2019 Dec 18;12(1):10. doi: 10.3390/cancers12010010 (PMC7016534; doi:10.3390/cancers12010010)
Supplement: Supplementary file 1 [file cancers-12-00010-s001.zip › Figure supplementary 1.pdf]

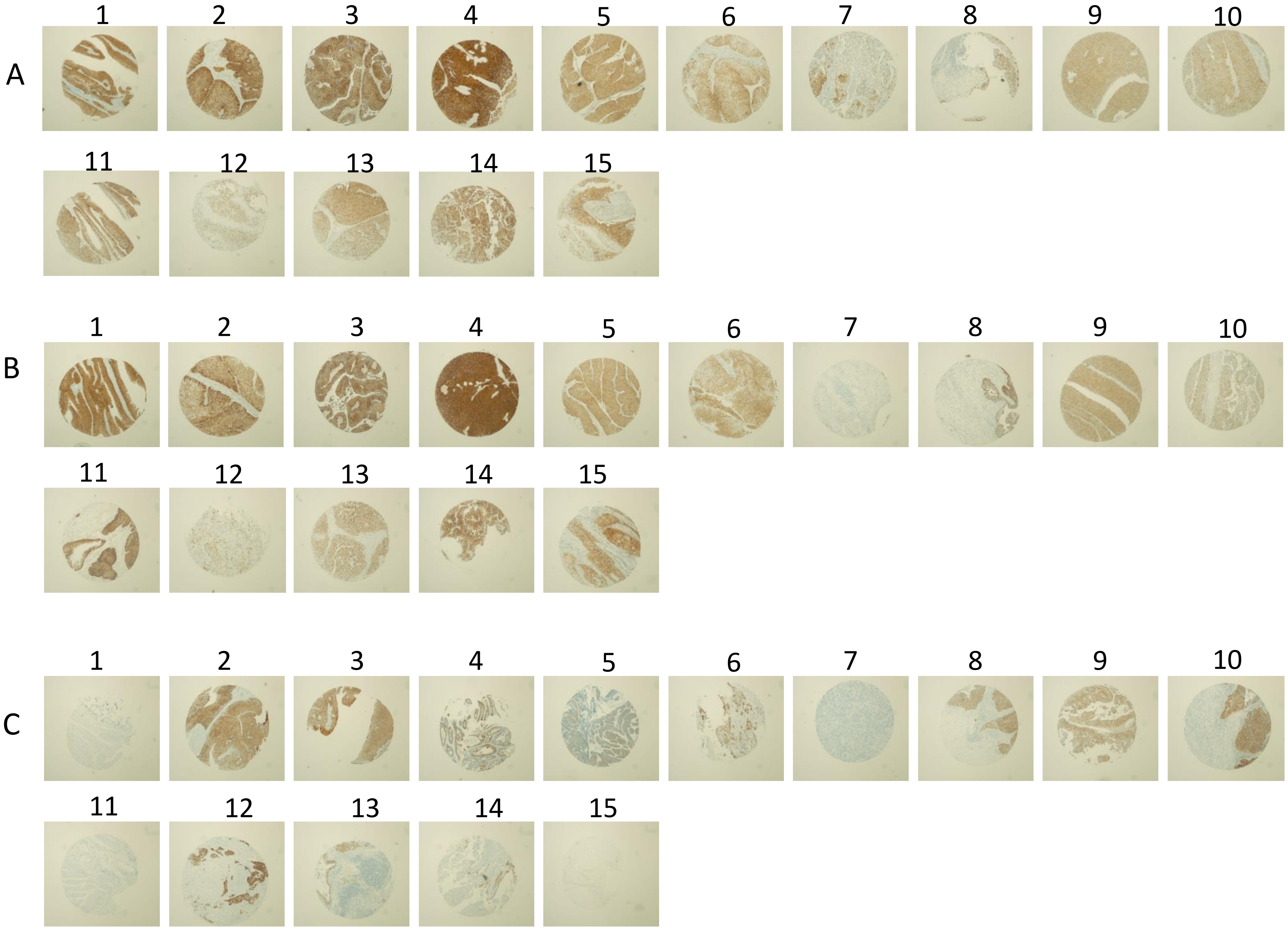
A
1 2 3 4 5 6 7 8 9 10
11 12 13 14 15
B
1 2 3 4 5 6 7 8 9 10
11 12 13 14 15
C
1 2 3 4 5 6 7 8 9 10
11 12 13 14 15

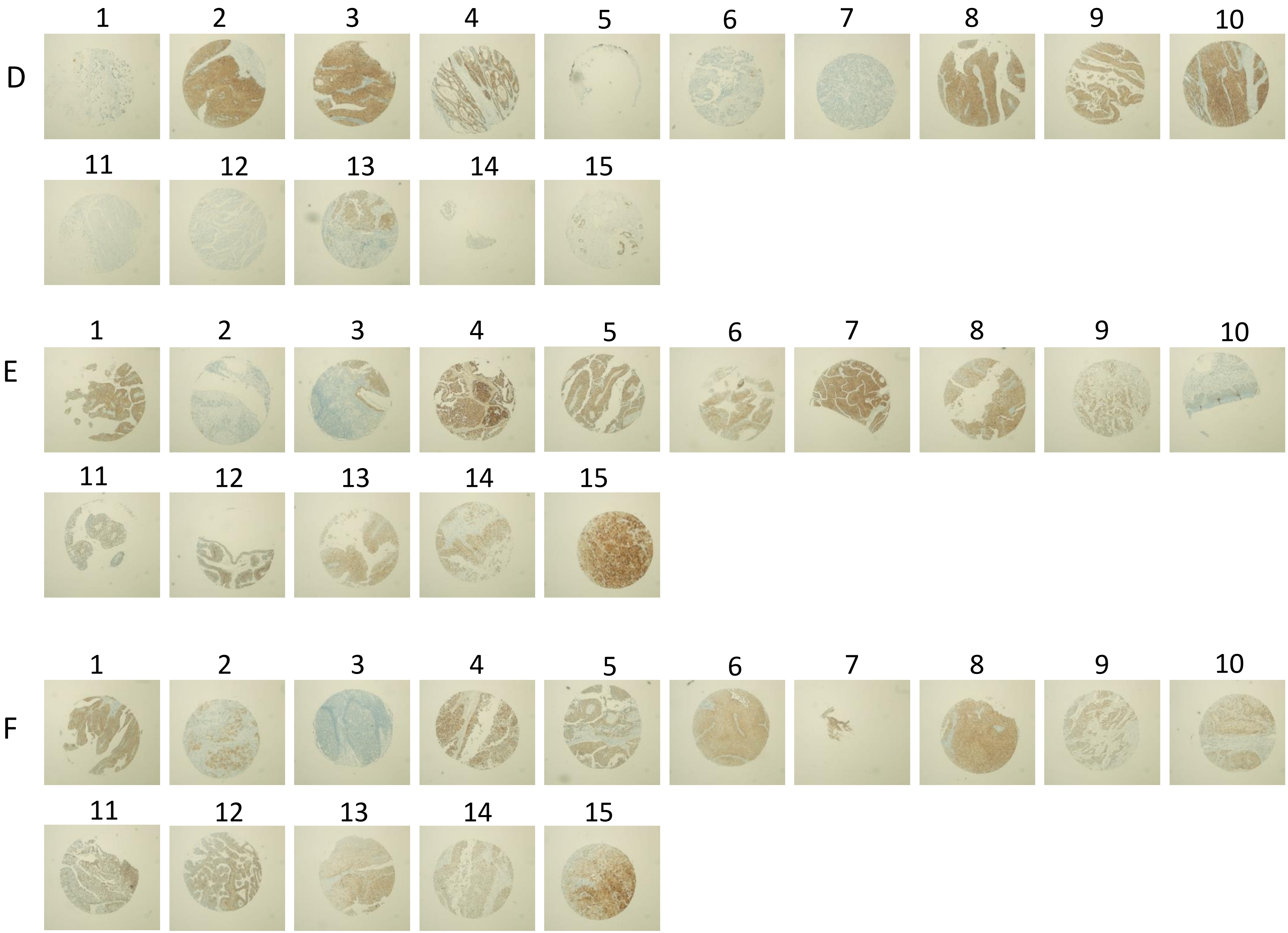
D
1 2 3 4 5 6 7 8 9 10
11 12 13 14 15
E
1 2 3 4 5 6 7 8 9 10
11 12 13 14 15
F
1 2 3 4 5 6 7 8 9 10
11 12 13 14 15

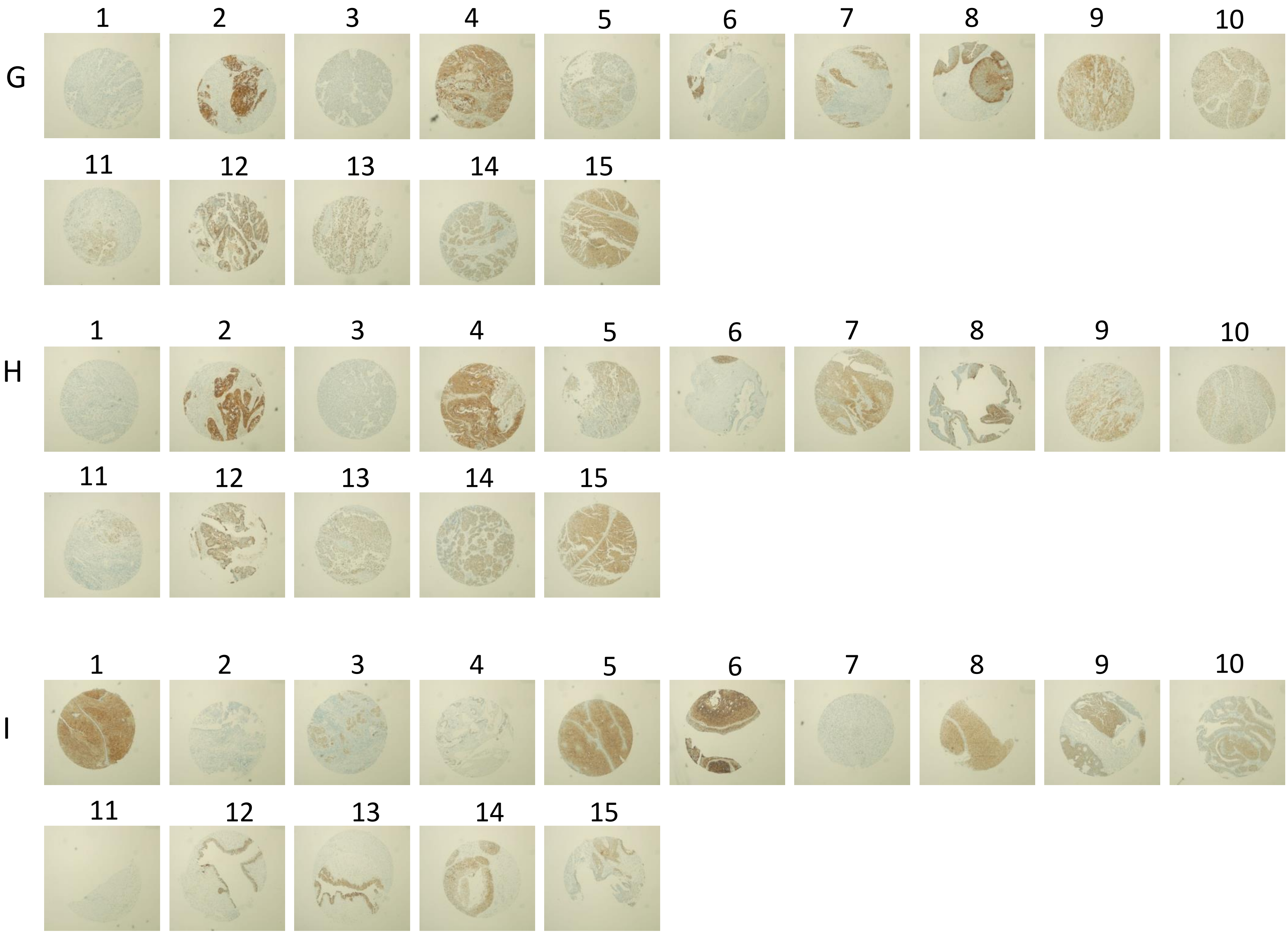
G
1 2 3 4 5 6 7 8 9 10
11 12 13 14 15
H
1 2 3 4 5 6 7 8 9 10
11 12 13 14 15
I
1 2 3 4 5 6 7 8 9 10
11 12 13 14 15

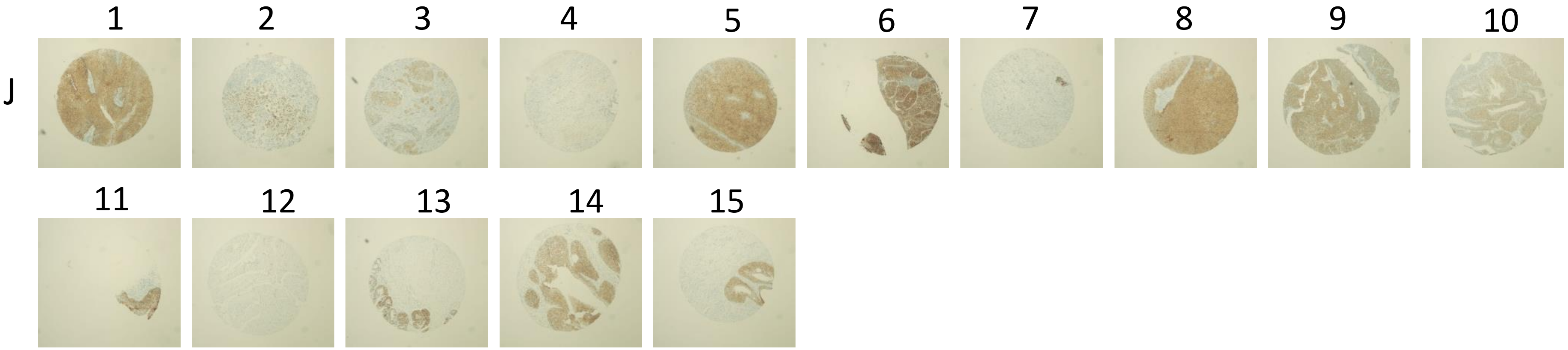

T-0:A1-5;
T-I:A6, A7, A15, B1, B6, B8, C2, C3, C5, C8, C11, C13, D7, E5, E11, E12
T-II: A8-11,A14,B2,B5,B7,B11,,B12,C1,C2,C4,C9,C10,C12,D2,D6,D8,D10,D11,D13,E1, E2,E6-10, E14
T-III:A12,A13,B3,B4,B9,C6,C7,C14,D3,D4,D5,D12,D14,E3,E4;

Figure Supplementary 1. IHC staining for maspin in human bladder tissue array with normal and bladder cancer tissues
