# Supplementary material for: Maspin is a PTEN-Upregulated and p53-Upregulated Tumor Suppressor Gene and Acts as an HDAC1 Inhibitor in Human Bladder Cancer
Source: Cancers (Basel). 2019 Dec 18;12(1):10. doi: 10.3390/cancers12010010 (PMC7016534; doi:10.3390/cancers12010010)
Supplement: Supplementary file 1 [file cancers-12-00010-s001.zip › Figure supplementary 2.pdf]

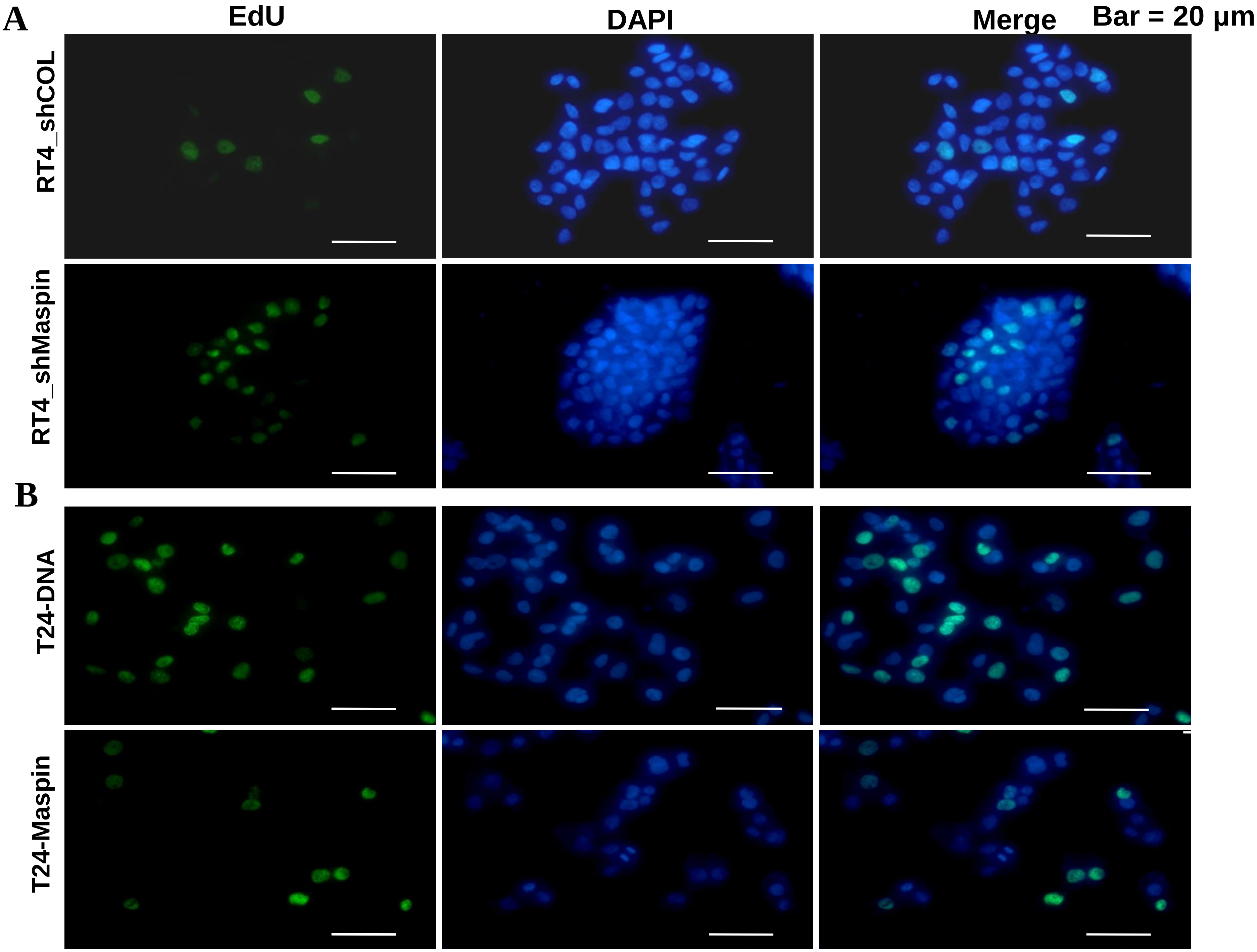

A
EdU
DAPI
Merge
Bar = 20 μm
RT4_shCOL
RT4_shMaspin
B
T24-DNA
T24-Maspin

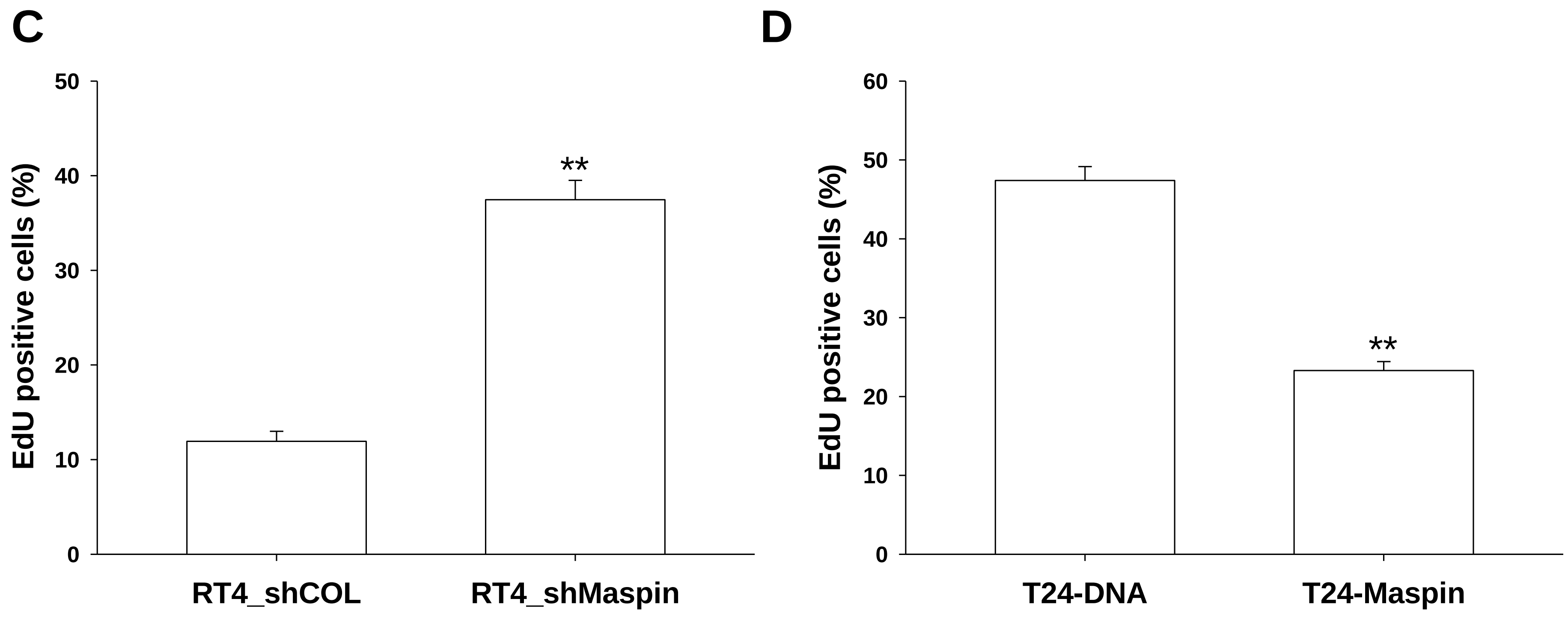

Figure Supplementary 2. The cell proliferation ability of RT4_shCOL, RT4_shMaspin (A, C), T24-DNA, and T24-Maspin cells (B, D) was determined using the EdU staining proliferation assay (± SE, n = 4).
