# Supplementary material for: Maspin is a PTEN-Upregulated and p53-Upregulated Tumor Suppressor Gene and Acts as an HDAC1 Inhibitor in Human Bladder Cancer
Source: Cancers (Basel). 2019 Dec 18;12(1):10. doi: 10.3390/cancers12010010 (PMC7016534; doi:10.3390/cancers12010010)
Supplement: Supplementary file 1 [file cancers-12-00010-s001.zip › Figure supplementary 3.pdf]

Fig 1E. RT4, HT, T24, 8301

Actin

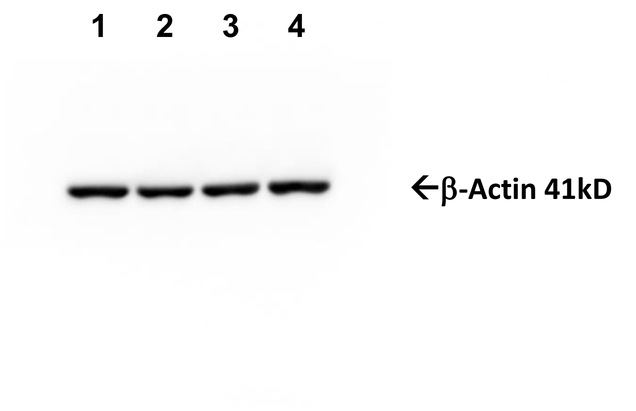

1: RT
2: HT1376
3: T24
4: TSGH-8301

Maspin

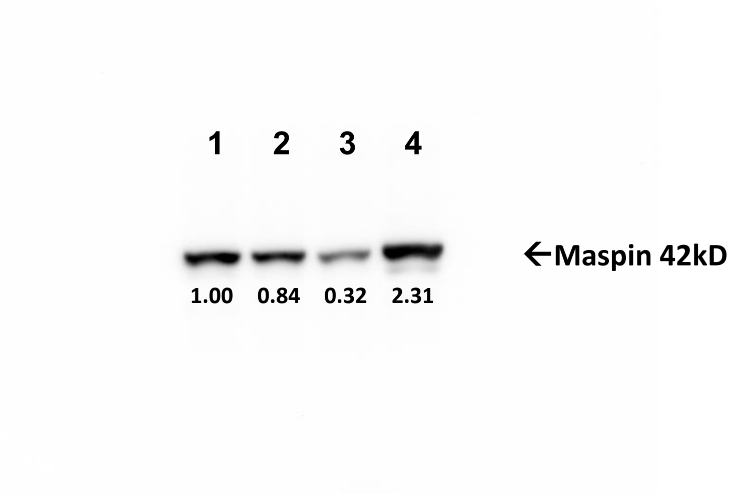

1: RT
2: HT1376
3: T24
4: TSGH-8301

Fig 2A. RT4(shCOL, shMaspin)

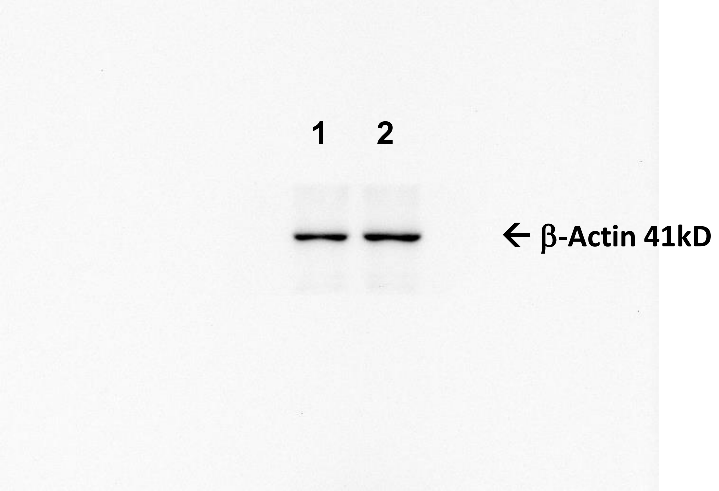

1: RT4_shCOL
2: RT4_shMaspin

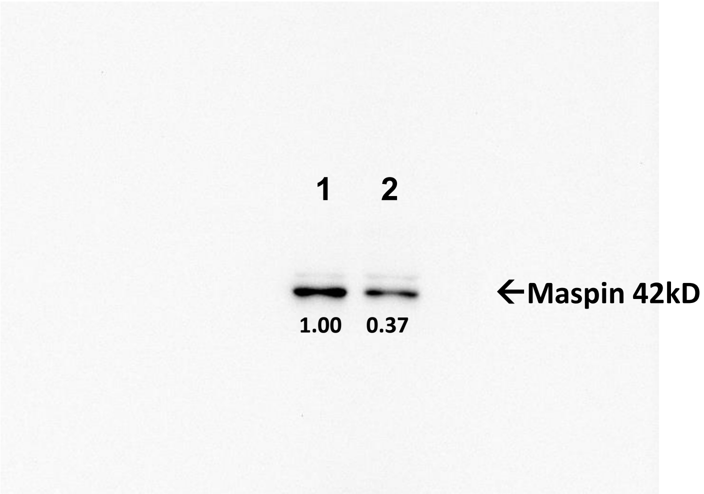

1: RT4-shCOL

2: RT4-shMaspin

Fig 2B. T24(DNA, MASPIN)

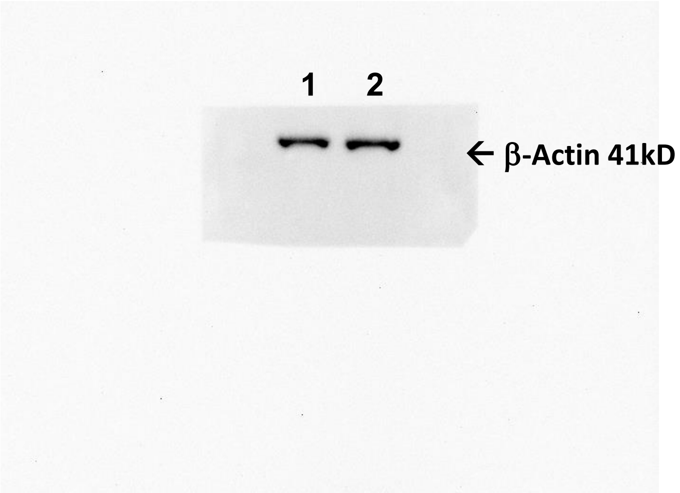

1: T24-DNA

2: T24-Maspin

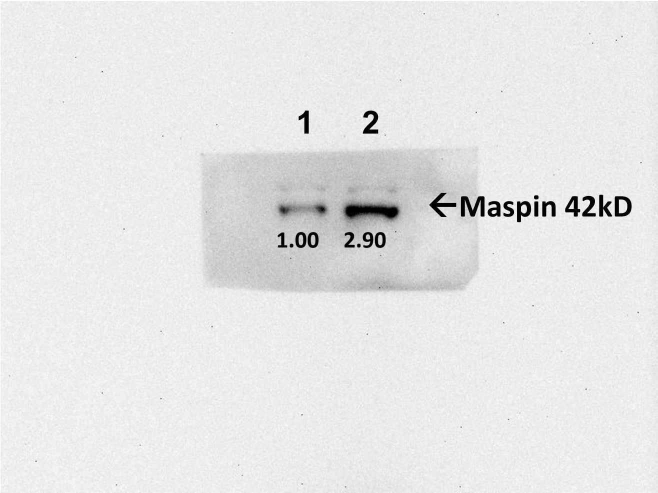

1: T24-DNA

2: T24-Maspin

Fig 3A. HT(shcoL, shMaspin)

Actin

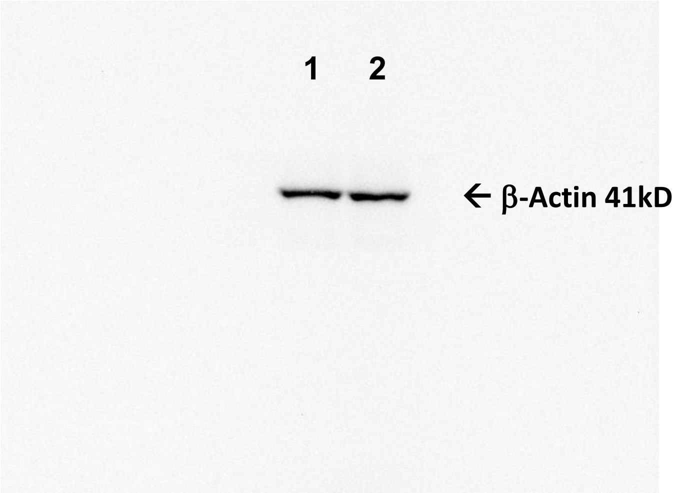

1: HT_shCOL

2: HT_shMaspin

Maspin

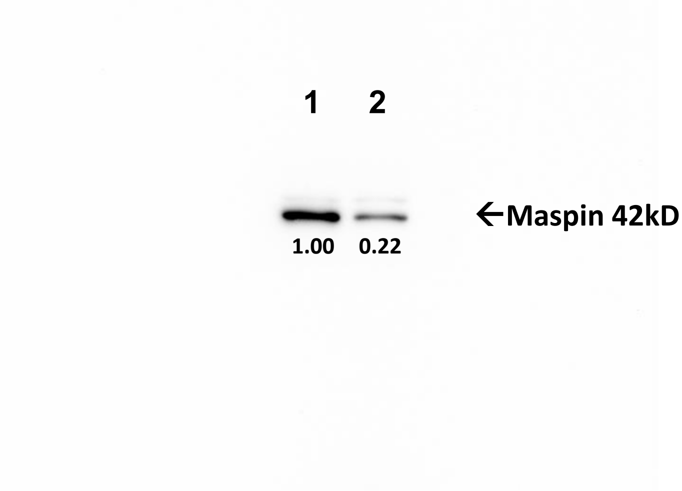

1: HT_shCOL

2: HT_shMaspin

Fig 3B. T24(DNA, MASPIN)

Actin

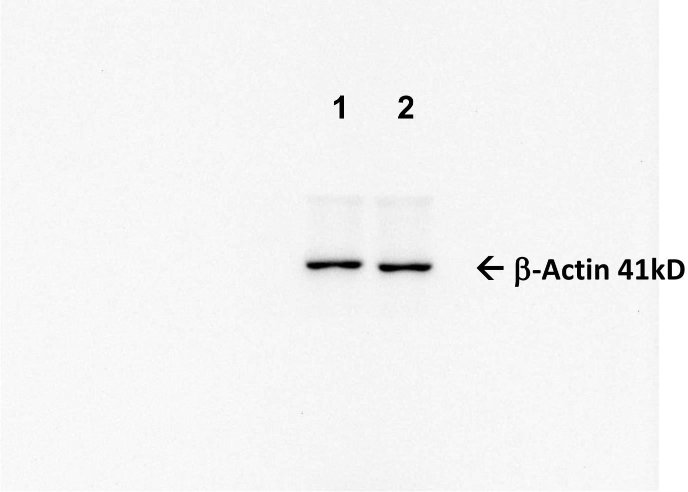

1: T24-DNA

2: T24-Maspin

Maspin

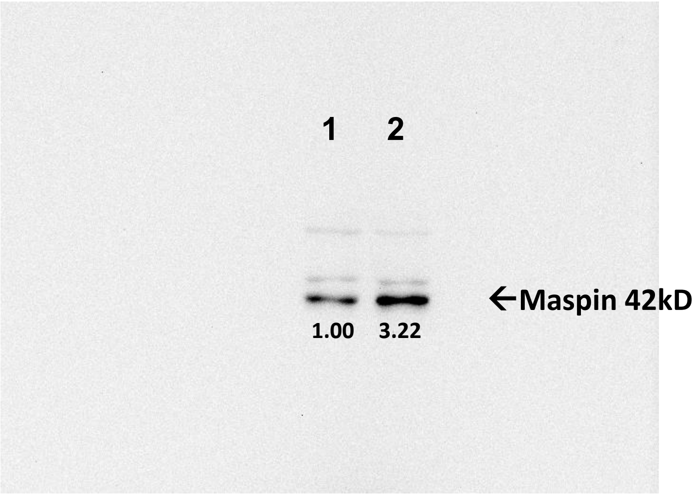

1: T24-DNA

2: T24-Maspin

Fig 4E. tumor xenografts HT(shCOL, shMaspin)

Actin

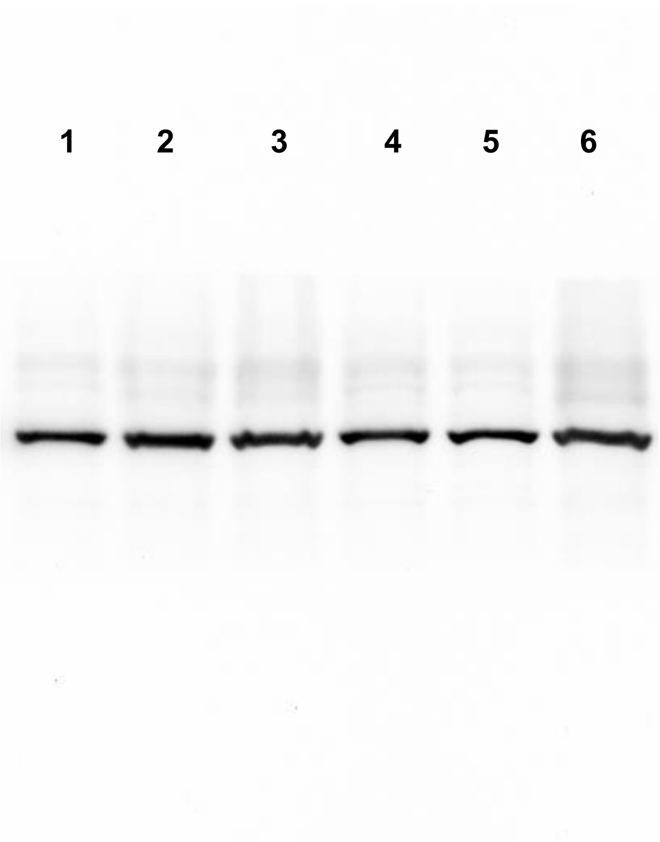

tumor xenografts

1~3: HT_shCOL

4~6: HT_shMaspin

←β-Actin 41kD

Maspin

tumor xenografts

1~3: HT_shCOL

4~6: HT_shMaspin

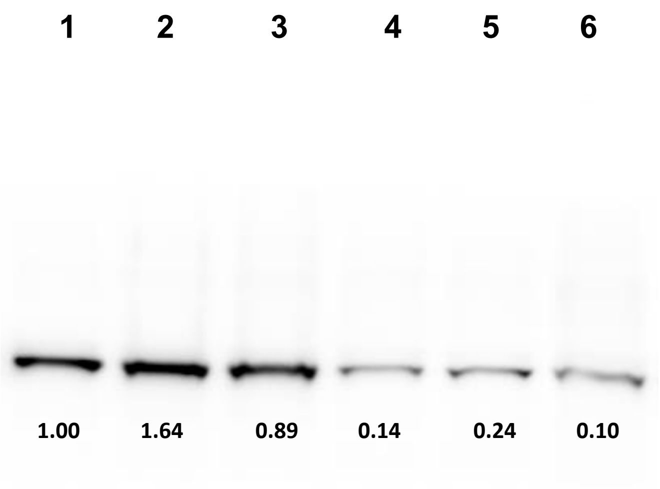

←Maspin 42kD

Fig 5A Left. RT4(shCOL, shMaspin)

Actin

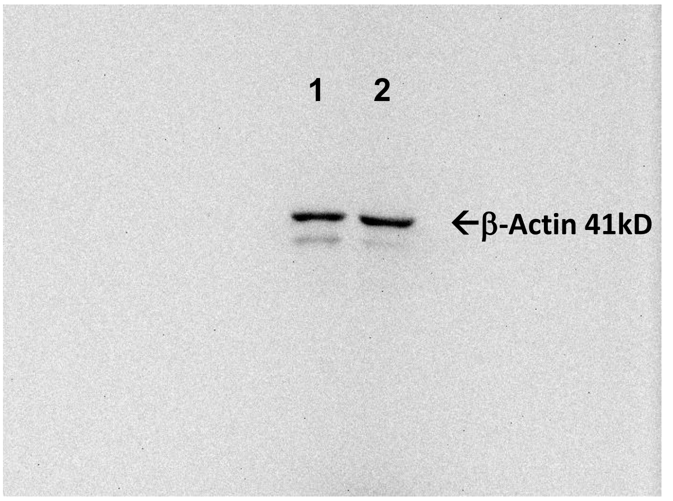

1: RT4_shCOL

2: RT4_shMaspin

Maspin

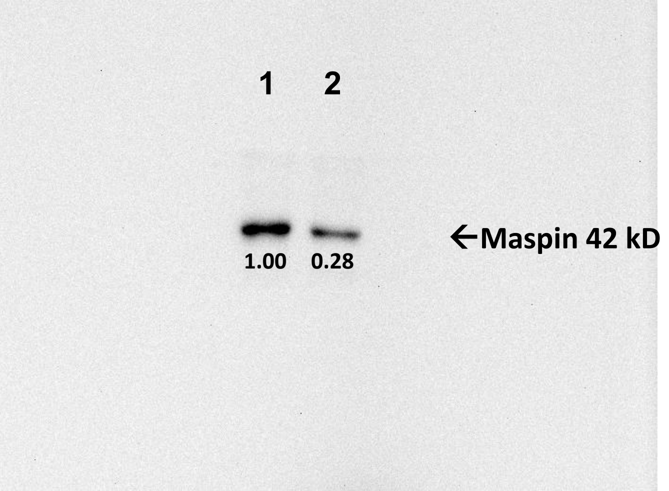

1: RT4_shCOL

2: RT4_shMaspin

Actin

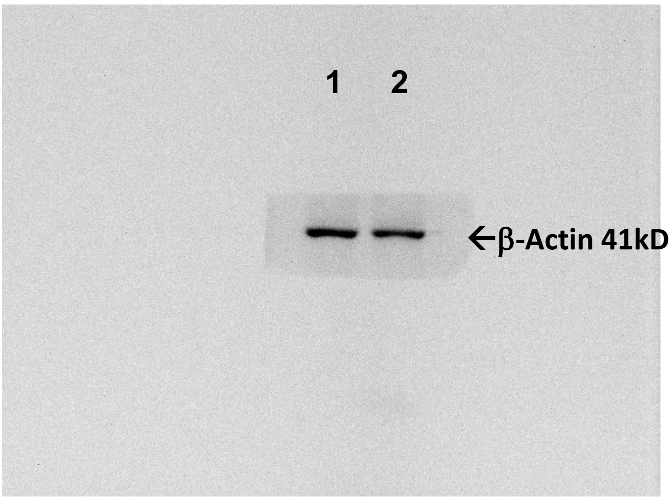

1: RT4_shCOL

2: RT4_shMaspin

Acetyl-H3

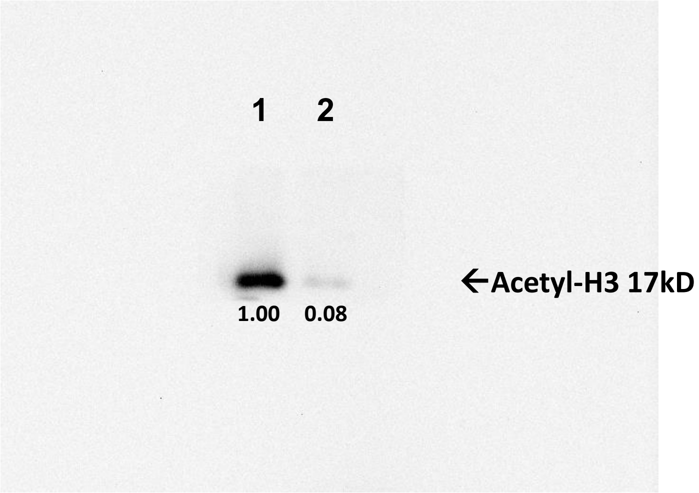

1: RT4_shCOL

2: RT4_shMaspin

Actin

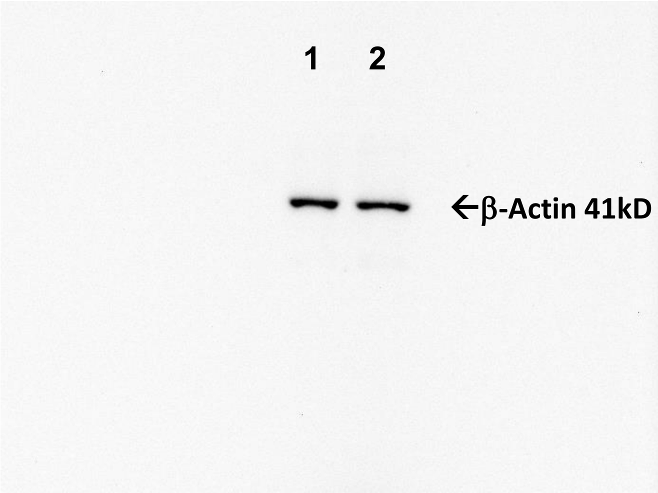

1: RT4_shCOL

2: RT4_shMaspin

HDAC1

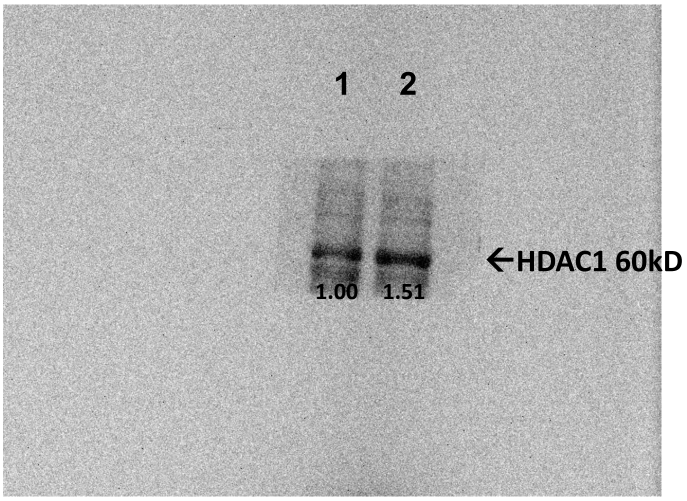

1: RT4_shCOL

2: RT4_shMaspin

Fig 5A Mid. HT(shCOL, shMaspin)

Actin

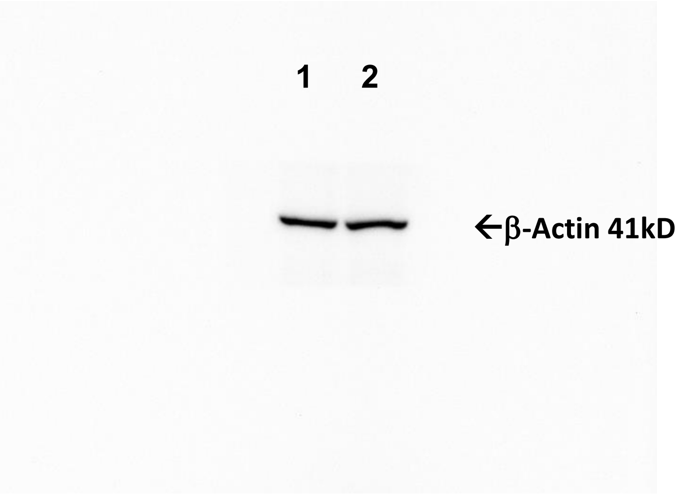

1: HT_shCOL

2: HT_shMaspin

Maspin

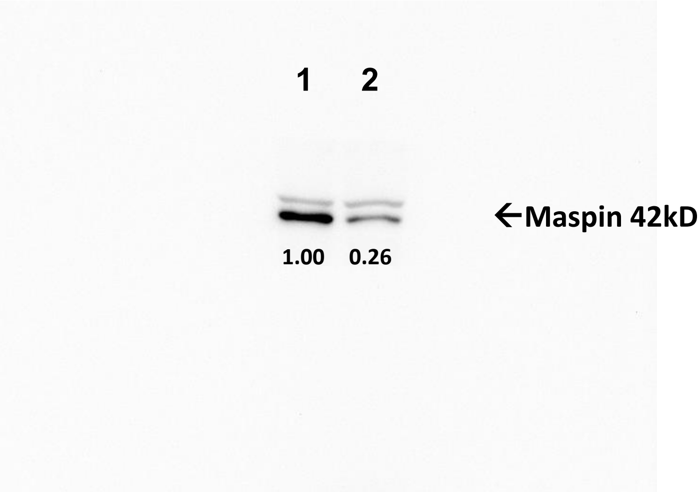

1: HT_shCOL

2: HT_shMaspin

Actin

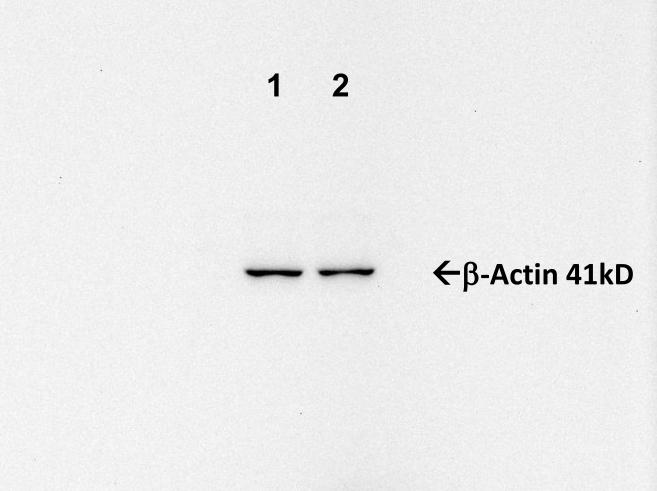

1: HT_shCOL

2: HT_shMaspin

Acetyl-H3

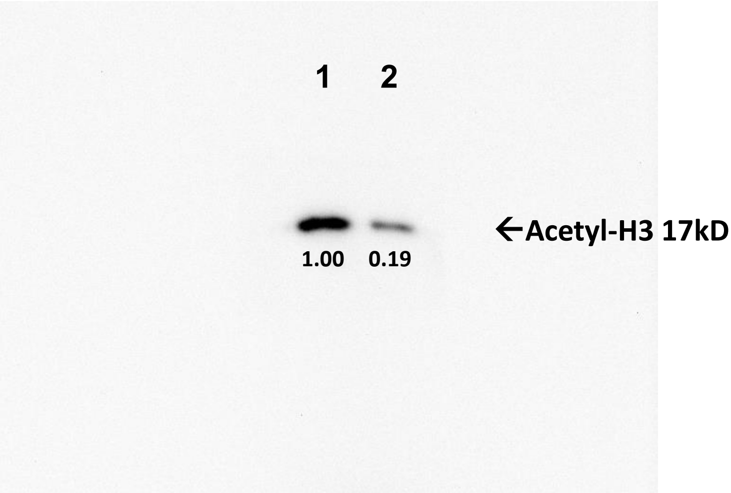

1: HT_shCOL
2: HT_shMaspin

Actin

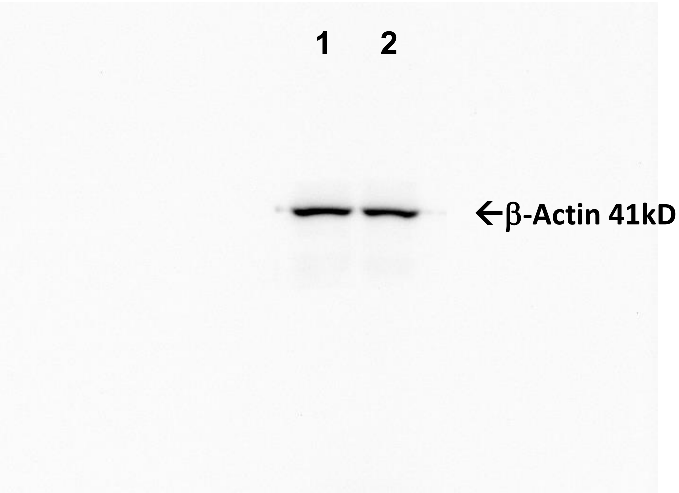

1: HT_shCOL
2: HT_shMaspin

HDAC1

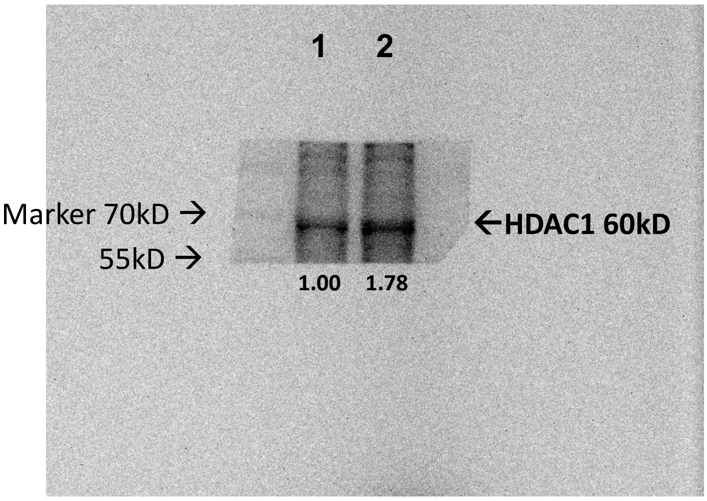

1: HT_shCOL
2: HT_shMaspin

Fig 5A Right. T24(DNA, Maspin)

Actin

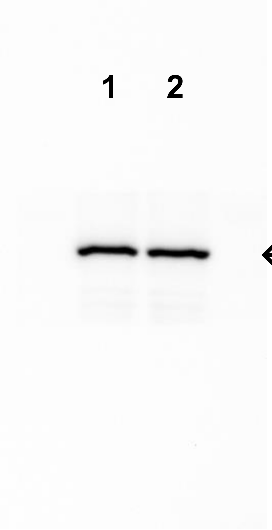

1: T24-DNA

2: T24-Maspin

Maspin

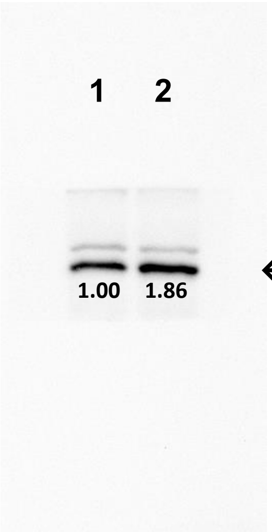

1: T24-DNA

2: T24-Maspin

Actin

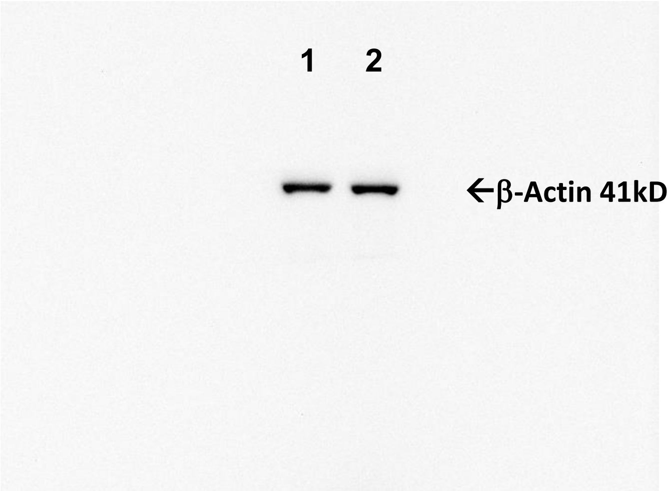

1: T24-DNA

2: T24-MASPIN

Acetyl-H3

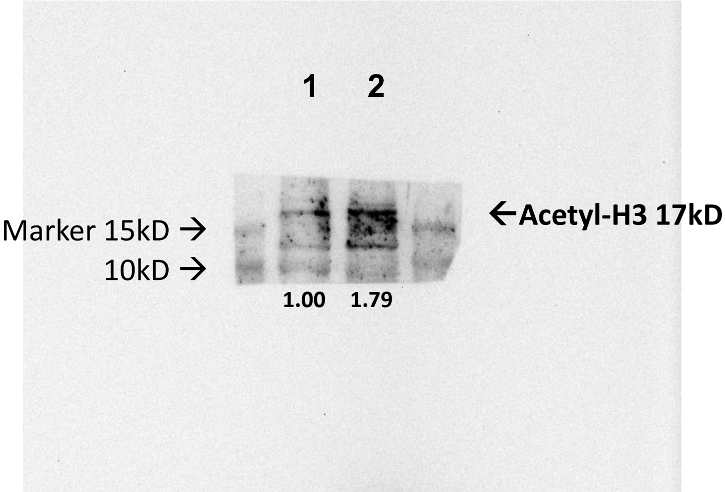

1: T24-DNA

2: T24-Maspin

Actin

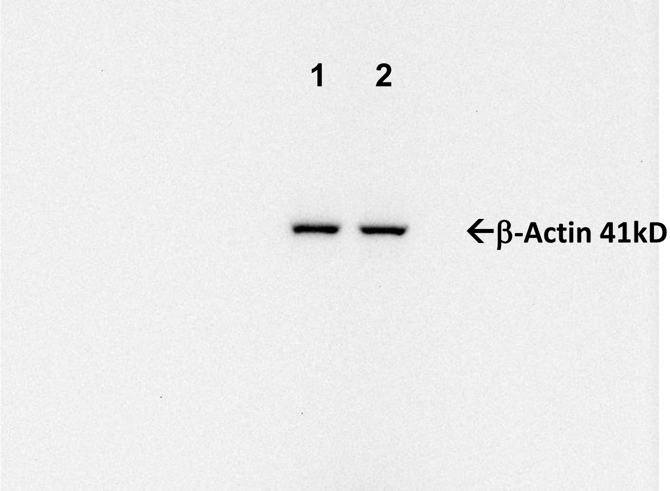

1: T24-DNA

2: T24-Maspin

HDAC1

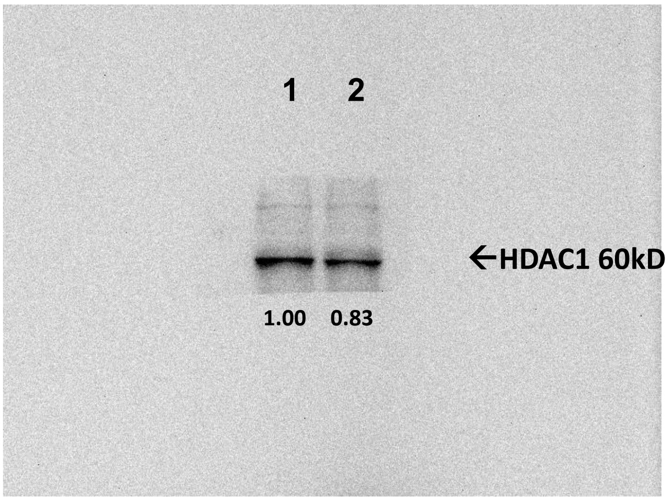

1: T24-DNA

2: T24-Maspin

Fig 5D Left. RT4(shCOL, shMaspin)

Actin

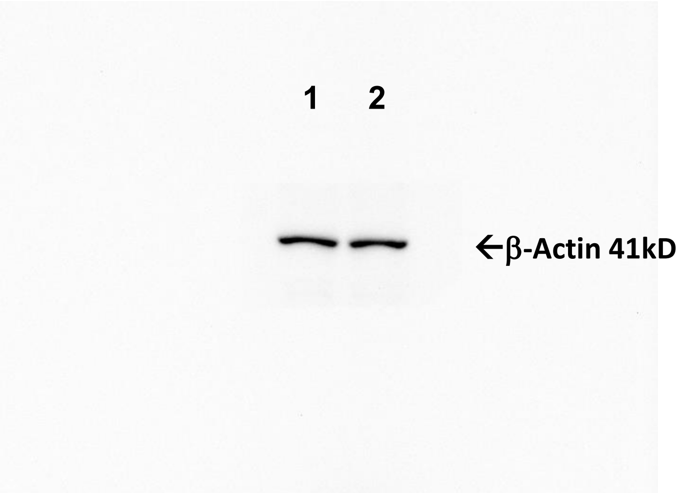

1: RT4_shCOL

2: RT4_shMaspin

Maspin

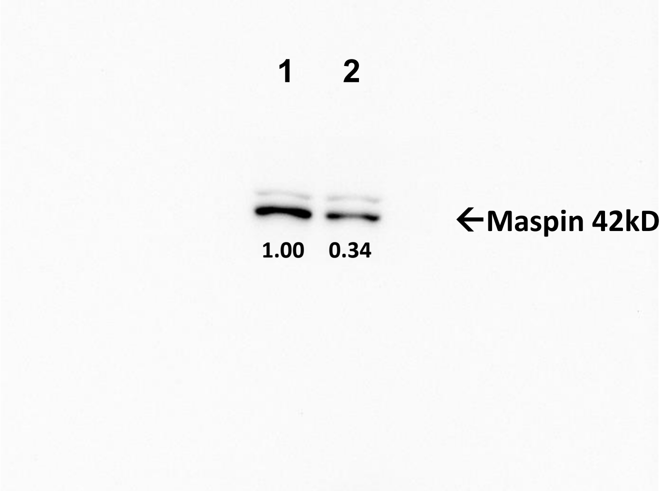

1: RT4_shCOL

2: RT4_shMaspin

CyclinD1

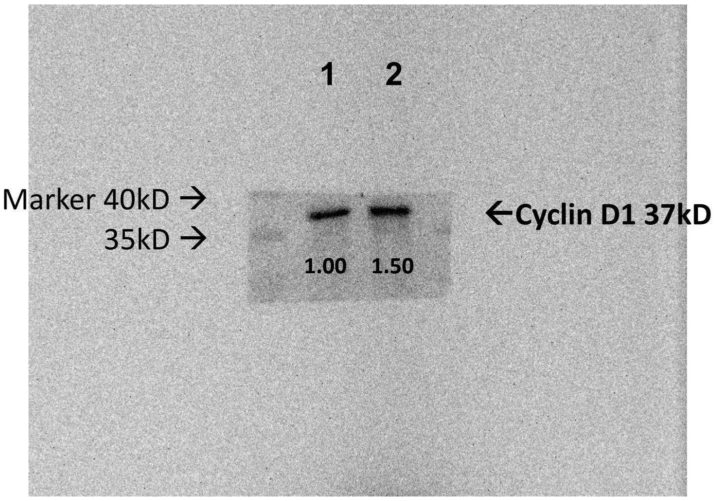

1: RT4_shCOL

2: RT4_shMaspin

P21

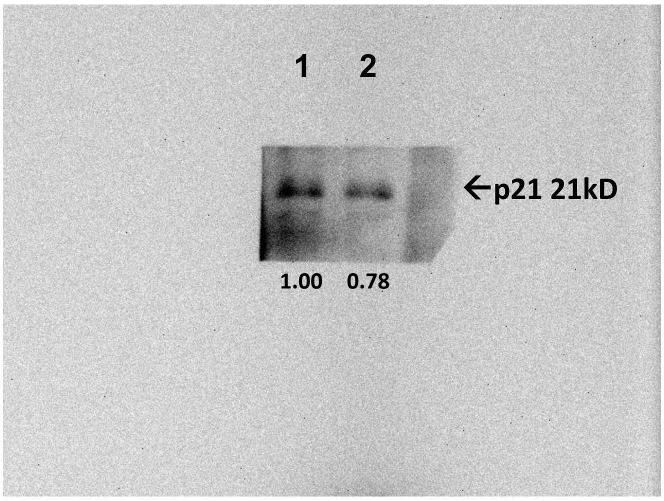

1: RT4_shCOL
2: RT4_shMaspin

Vimentin

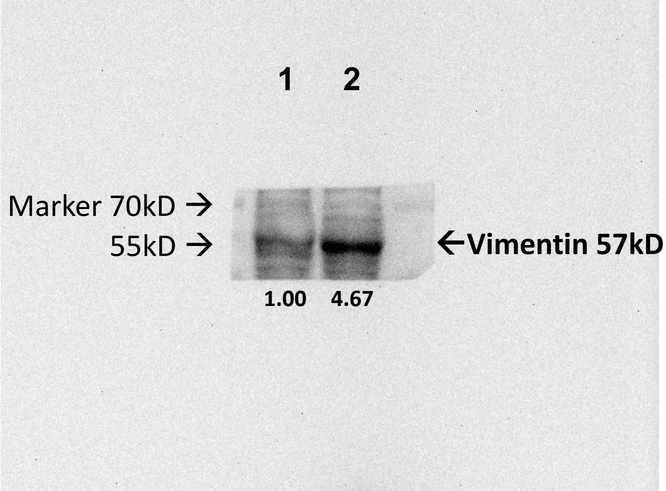

1: RT4_shCOL
2: RT4_shMaspin

MMP9

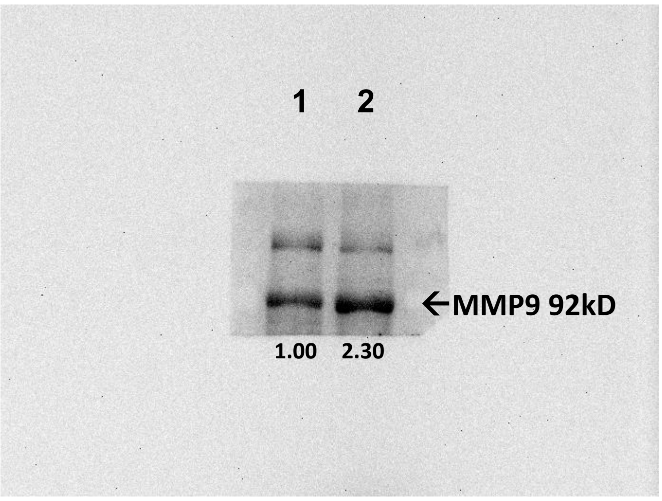

1: RT4_shCOL
2: RT4_shMaspin

Fig 5D Mid. HT(shCOL, shMaspin)

Actin

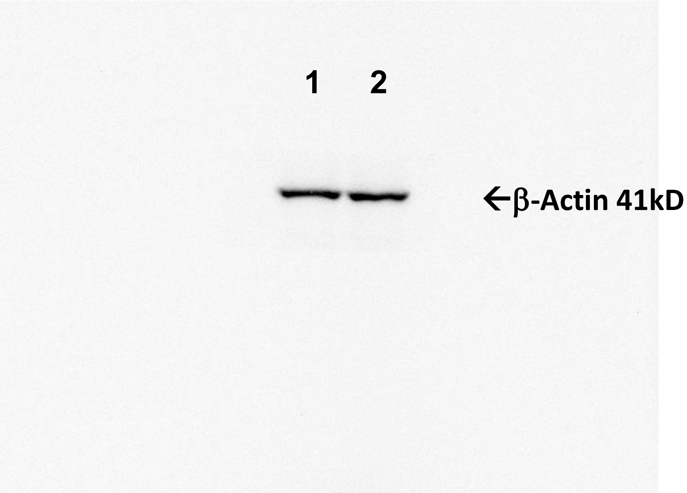

1: HT_shCOL

2: HT_shMaspin

Maspin

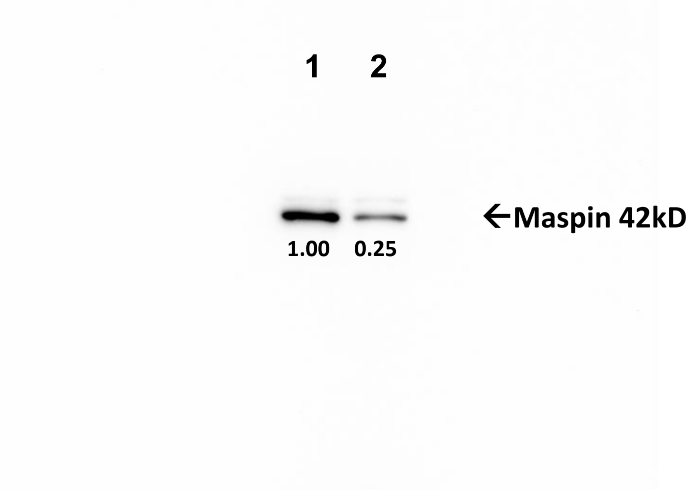

1: HT_shCOL

2: HT_shMaspin

CyclinD1

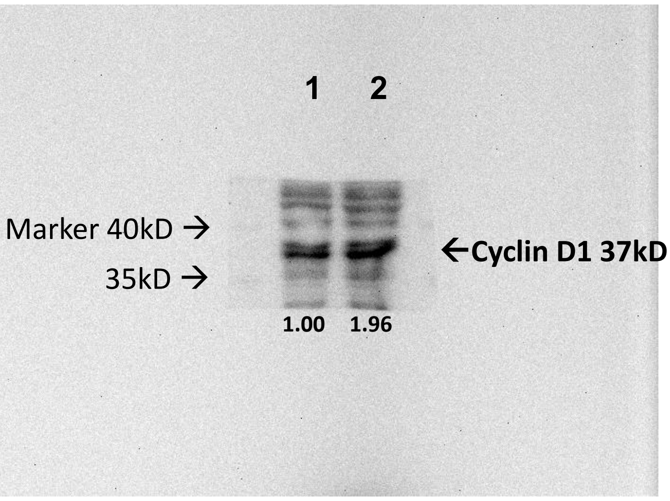

1: HT_shCOL

2: HT_shMaspin

P21

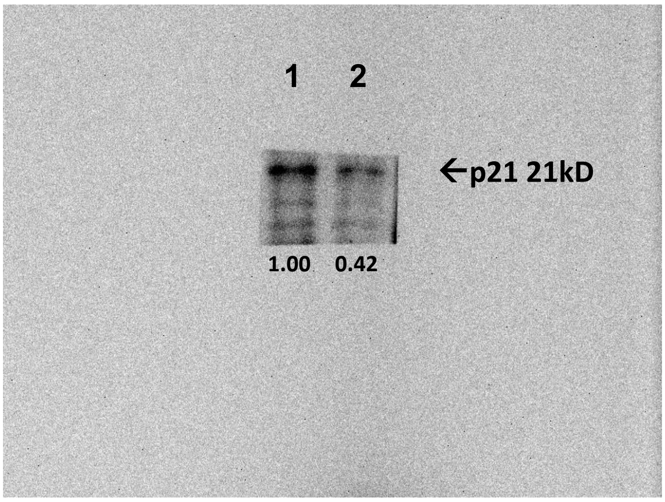

1: HT_shCOL

2: HT_shMaspin

Vimentin

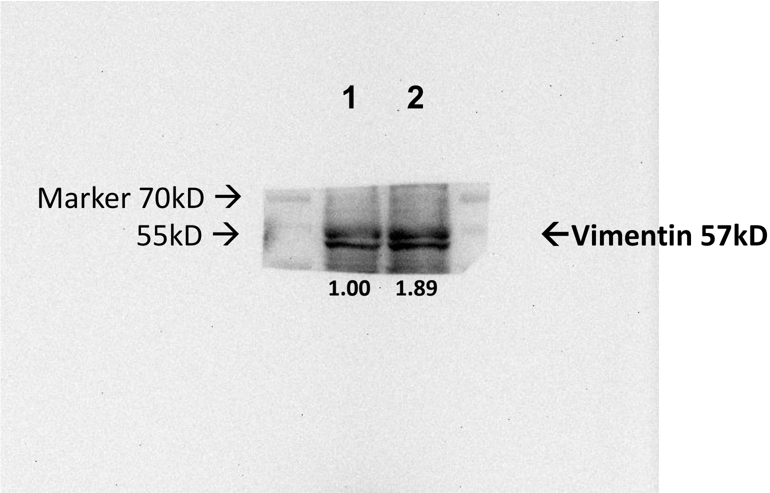

1: HT_shCOL

2: HT_shMaspin

MMP9

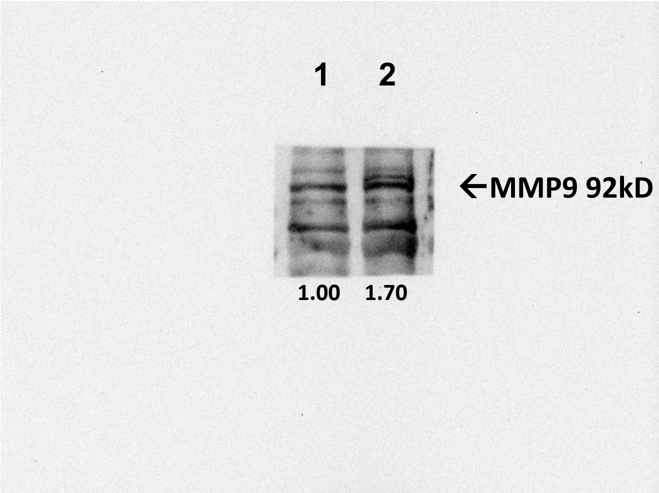

1: HT_shCOL

2: HT_shMaspin

Fig 5D Right. T24(DNA, Maspin)

Actin

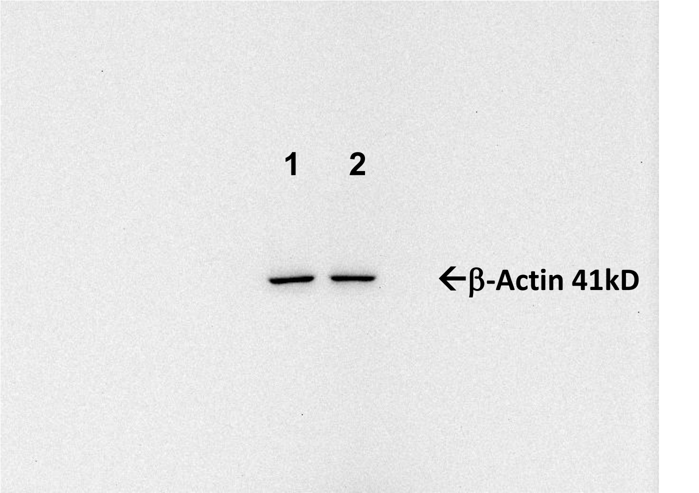

1: T24-DNA

2: T24-Maspin

Maspin

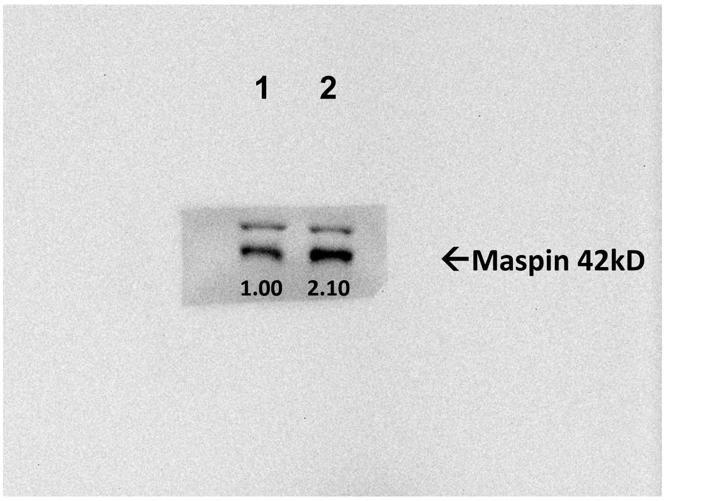

1: T24-DNA

2: T24-Maspin

CyclinD1

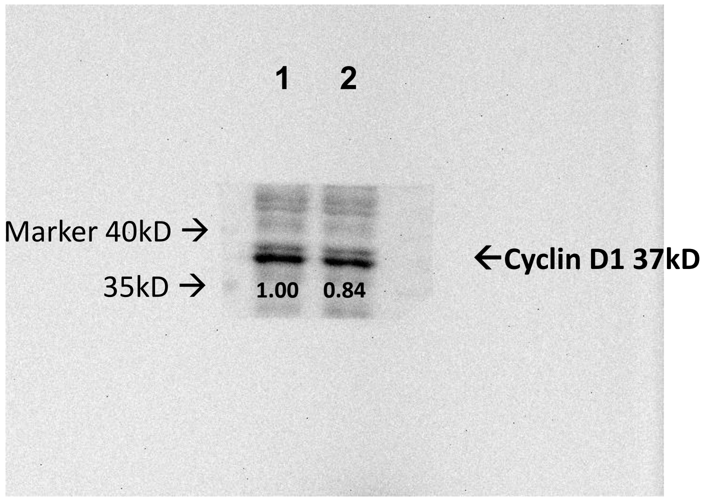

1: T24-DNA

2: T24-Maspin

P21

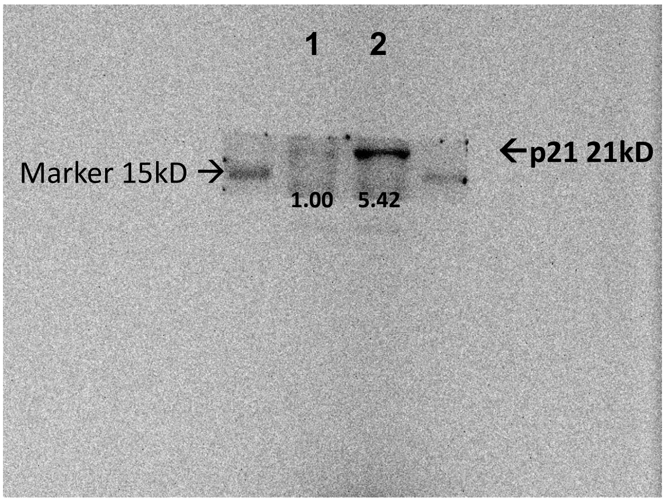

1: T24-DNA
2: T24-Maspin

Vimentin

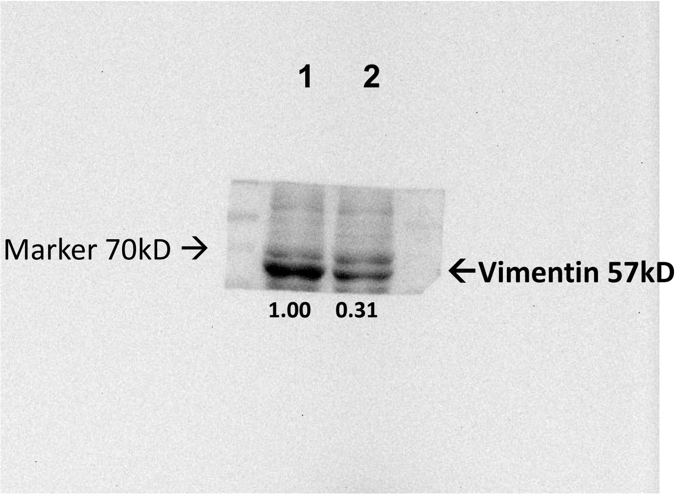

1: T24-DNA
2: T24-Maspin

MMP9

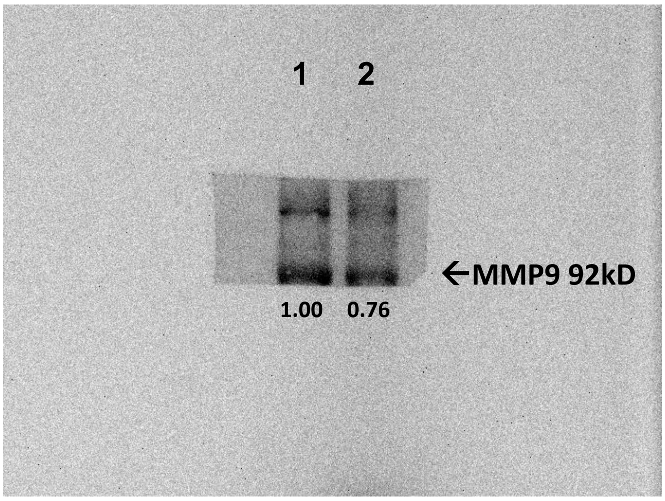

1: T24-DNA
2: T24-Maspin

Fig 6A Left. RT4(shCOL, shPTEN)

Actin

1 2

1: RT4_shCOL

2: RT4_shPTEN

←β-Actin 41kD

Maspin

1 2

1: RT4_shCOL

2: RT4_shPTEN

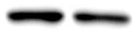

←Maspin 42kD

1.00 0.70

PTEN

1 2

1: RT4_shCOL

2: RT4_shPTEN

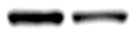

←PTEN 55kD

1.00 0.44

Fig 6A Right. T24(DNA, PTEN)

Actin

1 2

1: T24-DNA

2: T24-PTEN

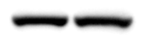

←β-Actin 41kD

Maspin

1 2

1: T24-DNA

2: T24-PTEN

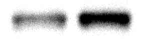

←Maspin 42kD

1.00 2.93

PTEN

1: T24-DNA

2: T24-PTEN

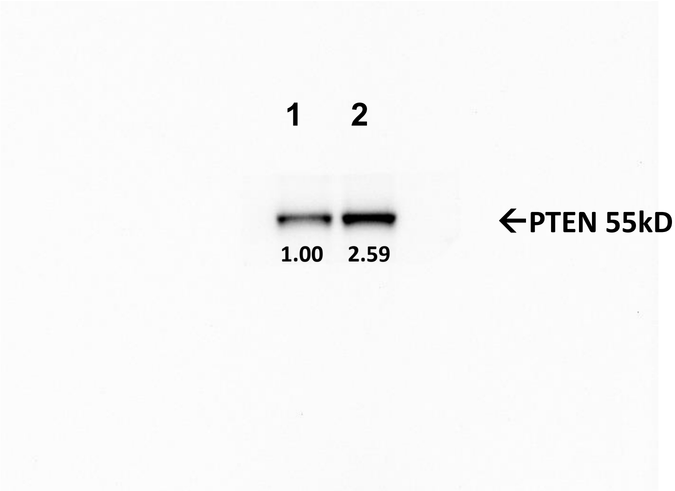

←PTEN 55kD

Fig 6E. RT4(control, shCOL, shPTEN)

Actin

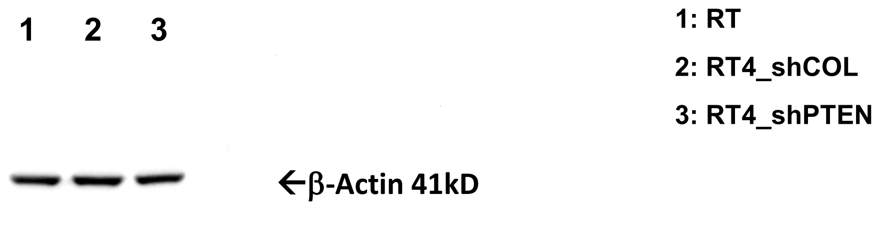

PTEN

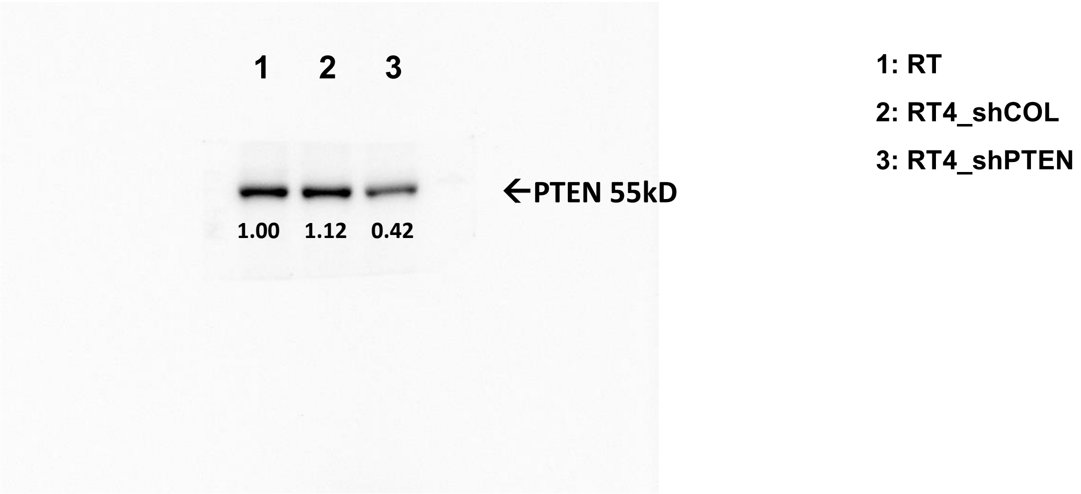

Maspin

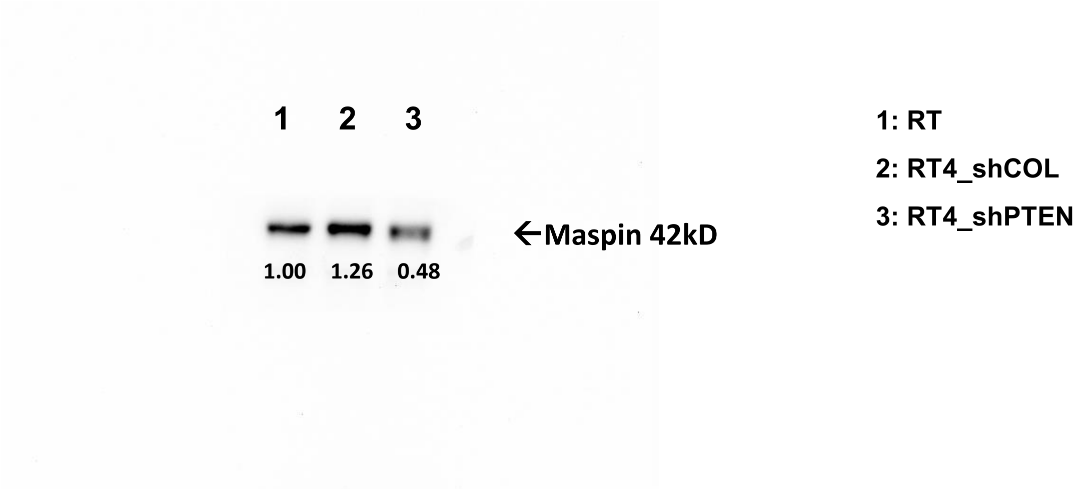

Akt

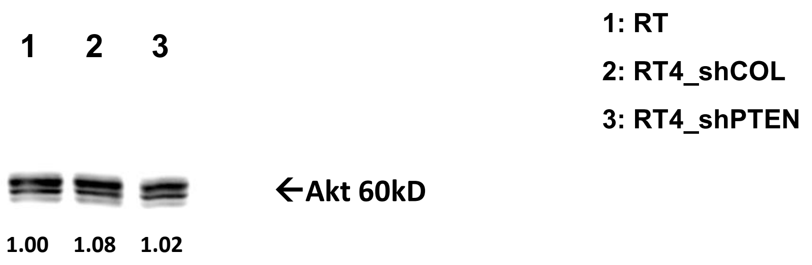

pAktS473

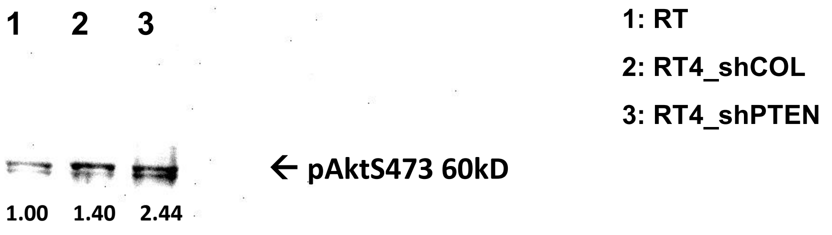

pAktT308

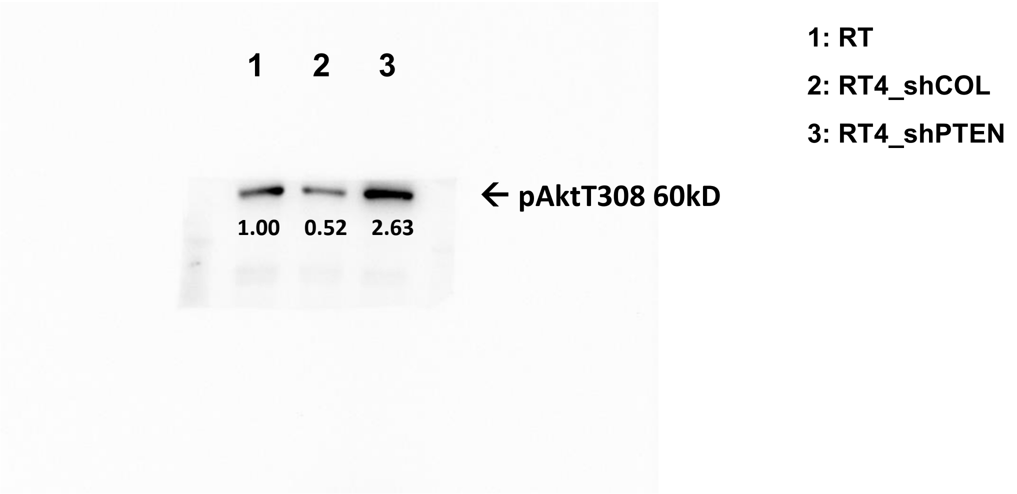

Fig 6F. RT4+VO-OHpic(0,250,500,1000)

Actin

1 2 3 4

1: RT+VO-OHpic 0 (uM)
2: RT+VO-OHpic 250
3: RT+VO-OHpic 500
4: RT+VO-OHpic 1000

←β-Actin 41kD

PTEN

1 2 3 4

1: RT+VO-OHpic 0 (uM)
2: RT+VO-OHpic 250
3: RT+VO-OHpic 500
4: RT+VO-OHpic 1000

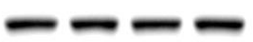

←PTEN 55kD

1.00 0.95 0.94 1.03

Maspin

1 2 3 4

1: RT+VO-OHpic 0 (uM)
2: RT+VO-OHpic 250
3: RT+VO-OHpic 500
4: RT+VO-OHpic 1000

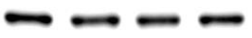

←Maspin 42kD

1.00 0.77 0.67 0.74

Akt

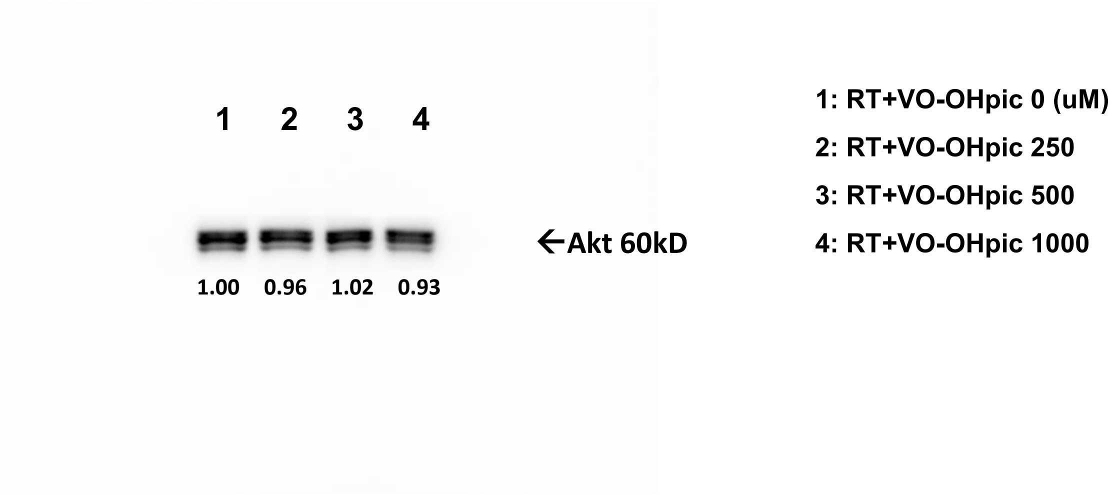

pAktS473

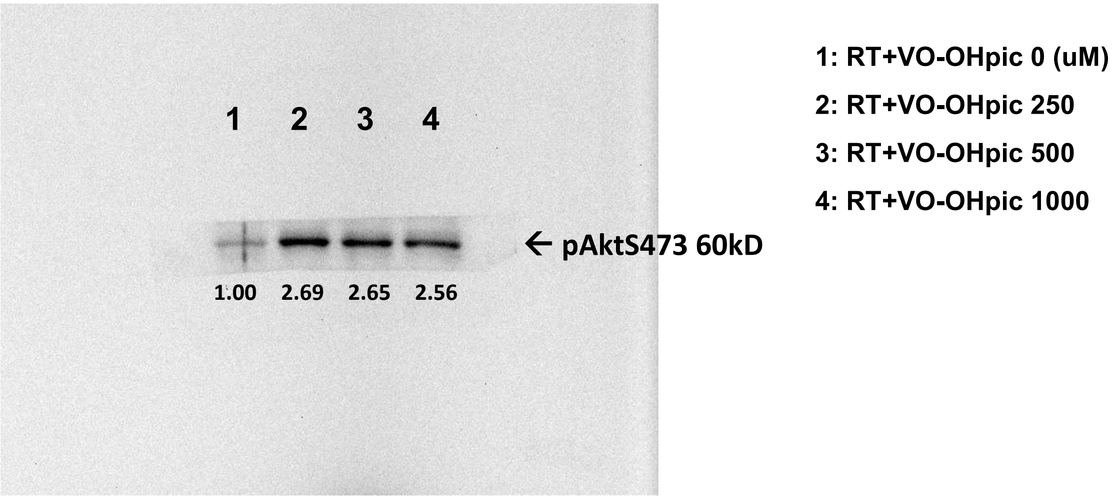

pAktT308

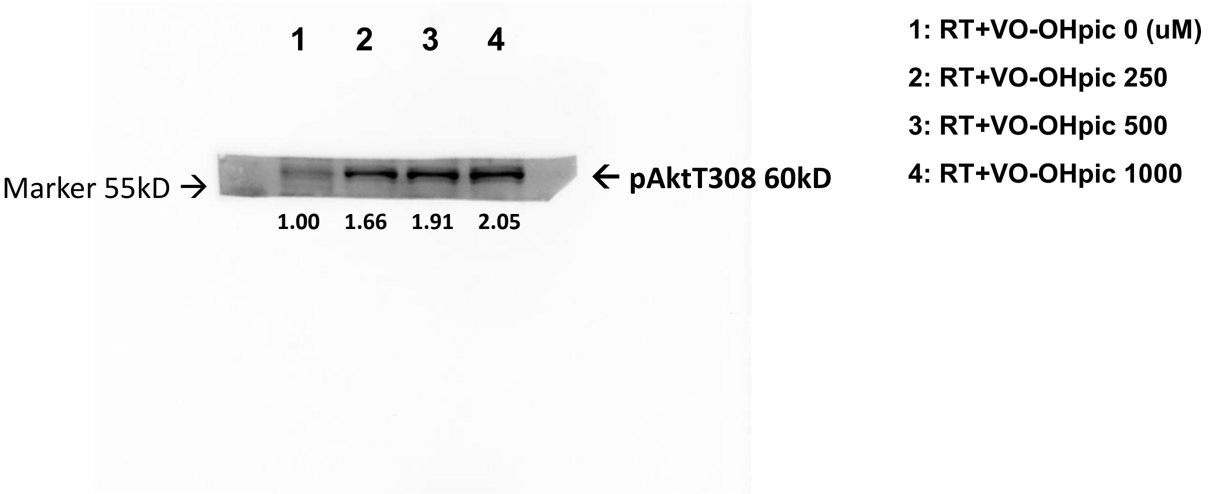

Fig 6I. Top. T24+MK2206(0,500,1000)

Actin

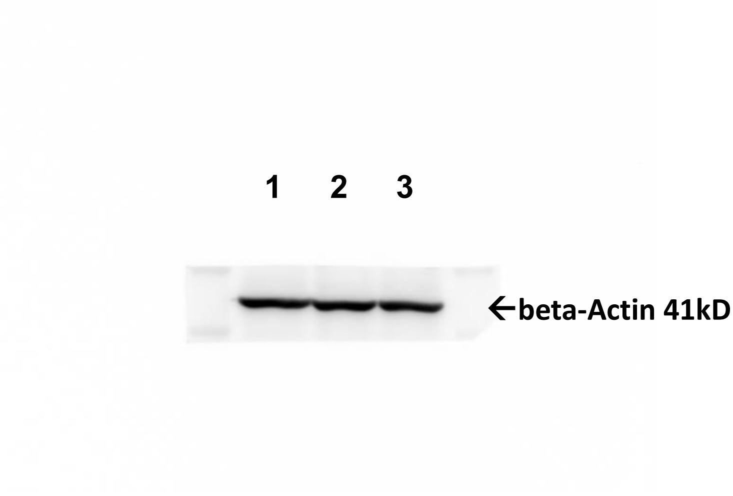

1: T24+MK2206 0 (uM)

2: T24+MK2206 500

3: T24+MK2206 1000

Akt

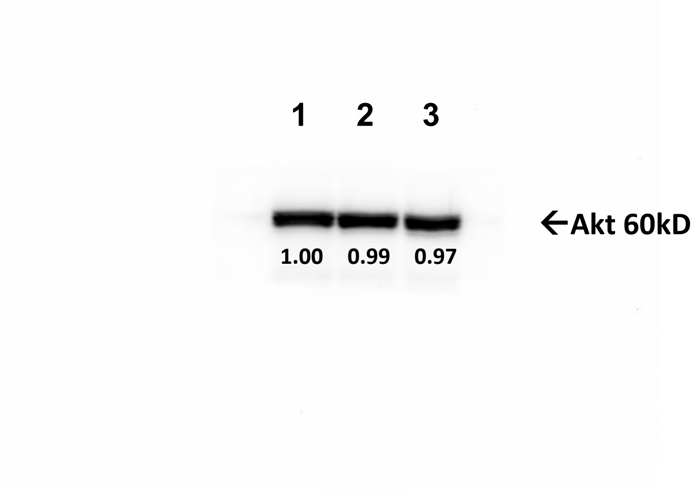

1: T24+MK2206 0 (uM)

2: T24+MK2206 500

3: T24+MK2206 1000

pAktS473

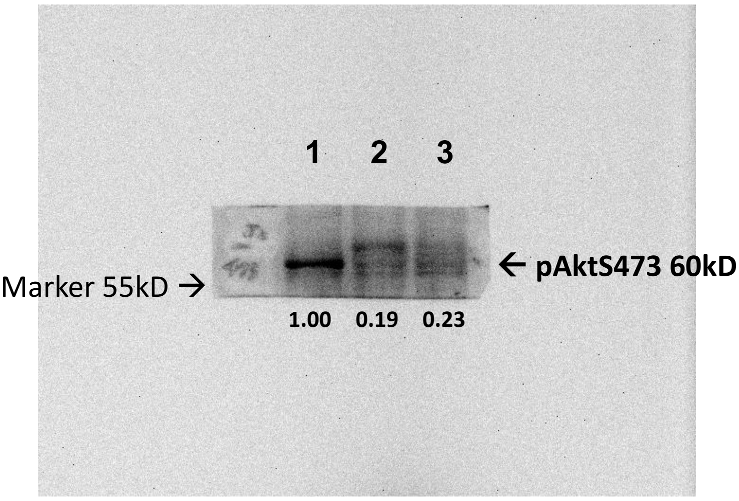

1: T24+MK2206 0 (uM)

2: T24+MK2206 500

3: T24+MK2206 1000

pAktT308

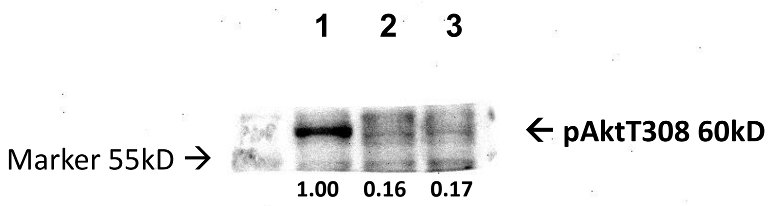

1: T24+MK2206 0 (uM)
2: T24+MK2206 500
3: T24+MK2206 1000

Fig 6I. bot. T24+MK2206(0,250,500,1000)
Actin

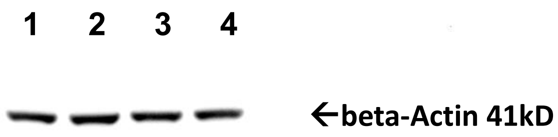

1: T24+MK2206 0 (uM)
2: T24+MK2206 250
3: T24+MK2206 500
4: T24+MK2206 1000

Maspin

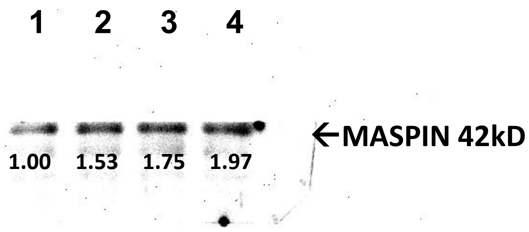

1: T24+MK2206 0 (uM)
2: T24+MK2206 250
3: T24+MK2206 500
4: T24+MK2206 1000

Fig 7A. HT(DNA, p53)

Actin

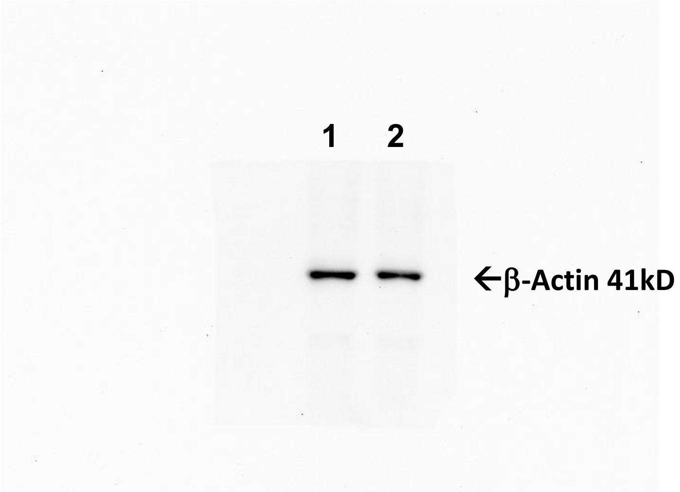

1: HT-DNA

2: HT-p53

p53

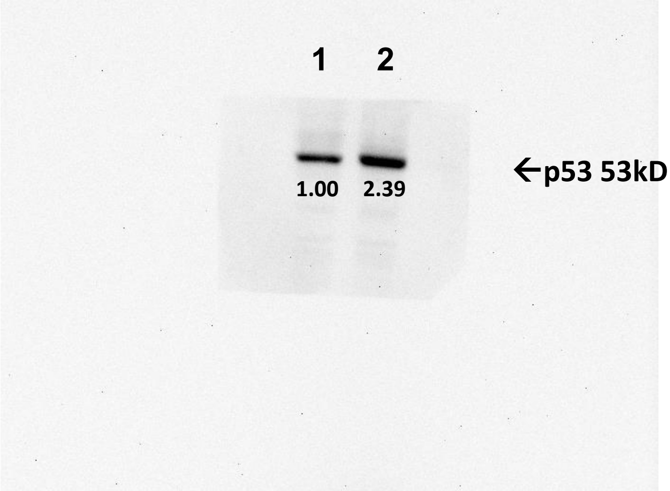

1: HT-DNA

2: HT-p53

Maspin

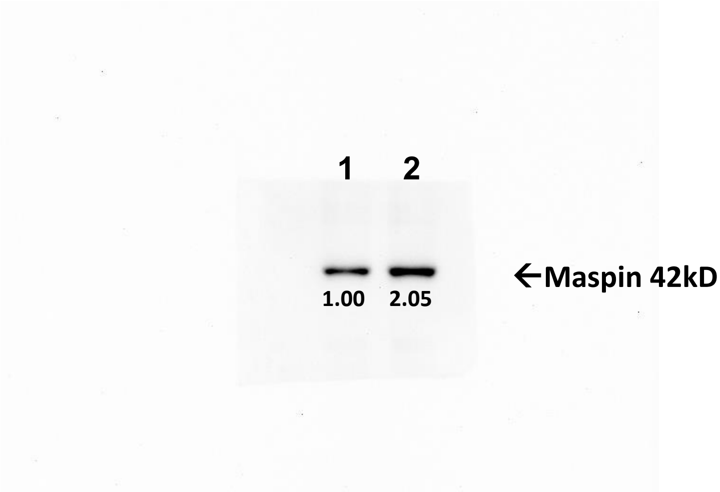

1: HT-DNA

2: HT-p53

Fig 7C. RT4_shCOL, RT4-shp53 ±Cpt

Actin

1: RT4_shCOL +Vehicle

2: RT4_hCOL +Cpt

3: RT4_shp53 +Vehicle

4: RT4_shp53 +Cpt

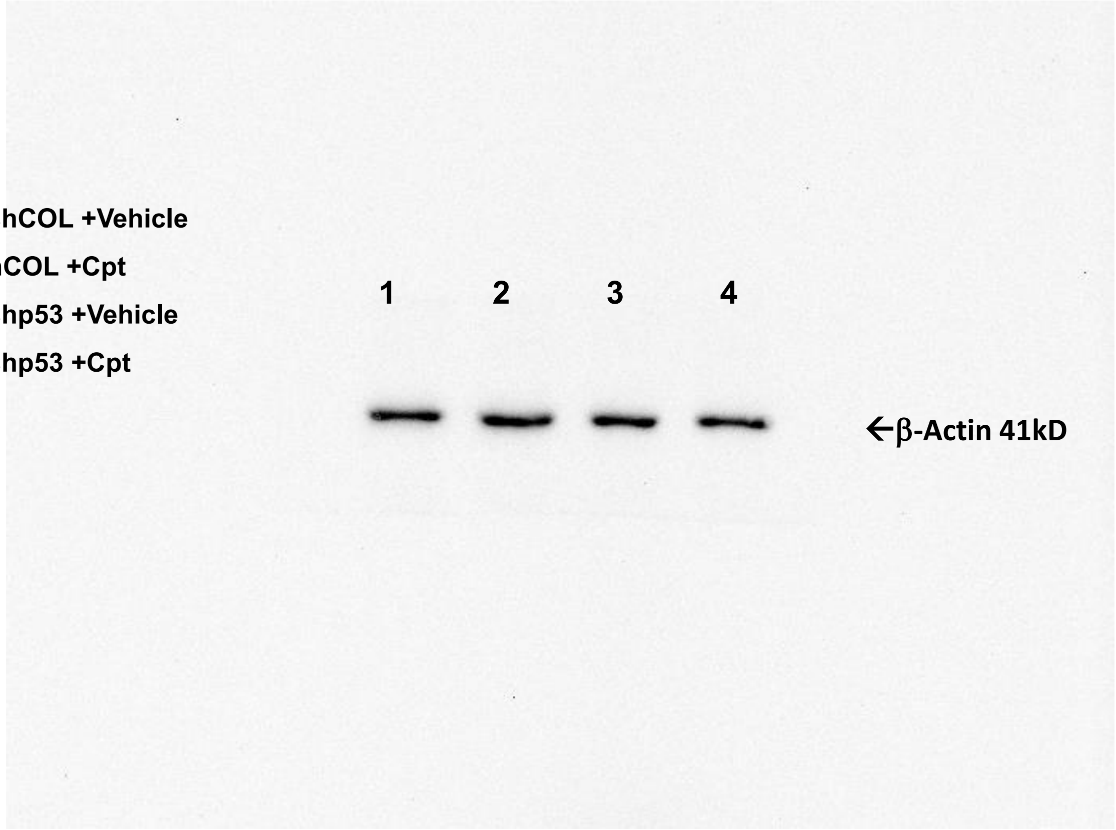

p53

1: RT4_shCOL +Vehicle

2: RT4_shCOL +Cpt

3: RT4_shp53 +Vehicle

4: RT4_shp53 +Cpt

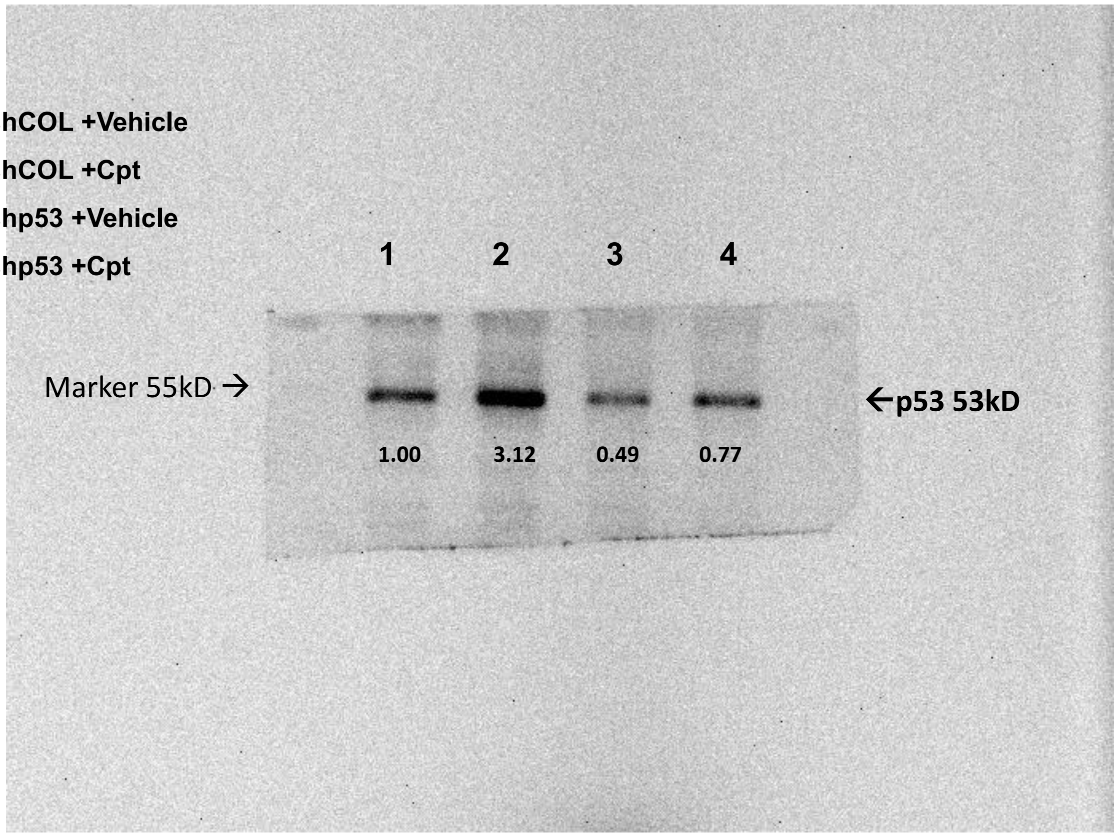

actin

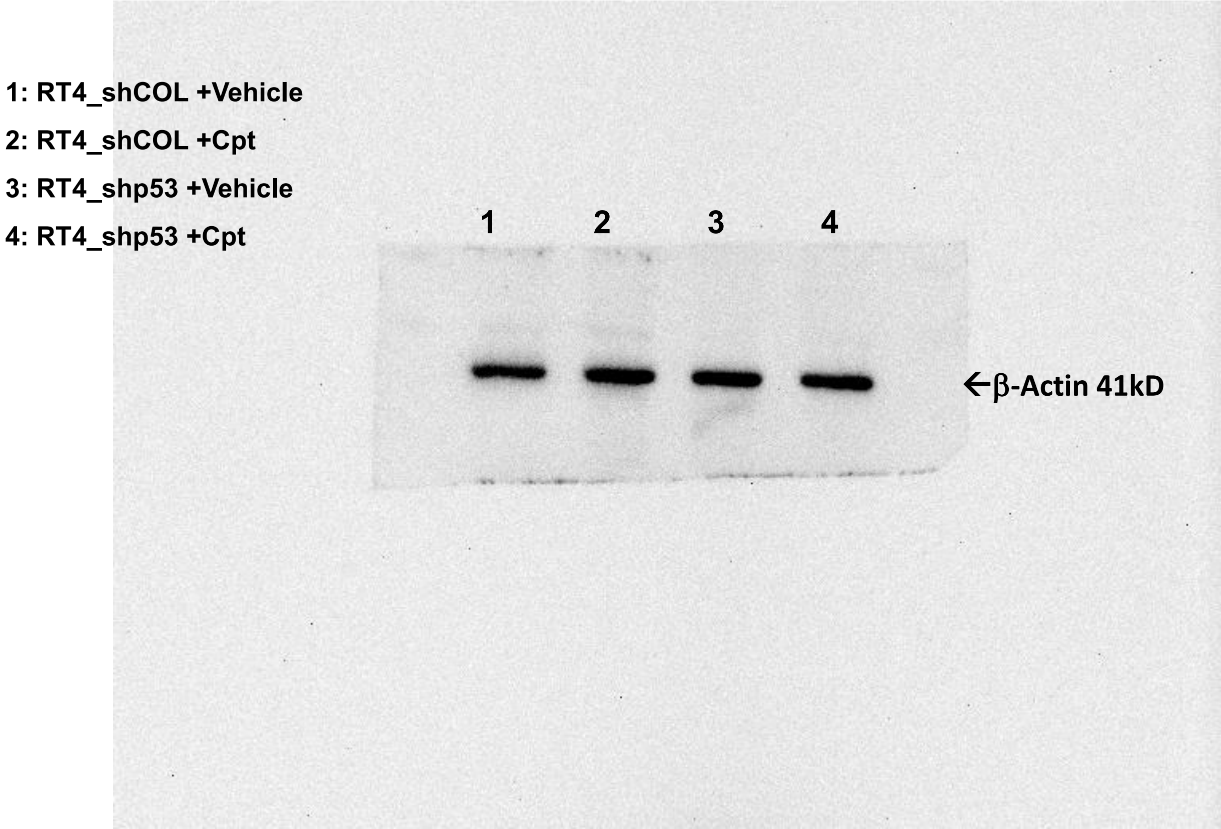

Maspin

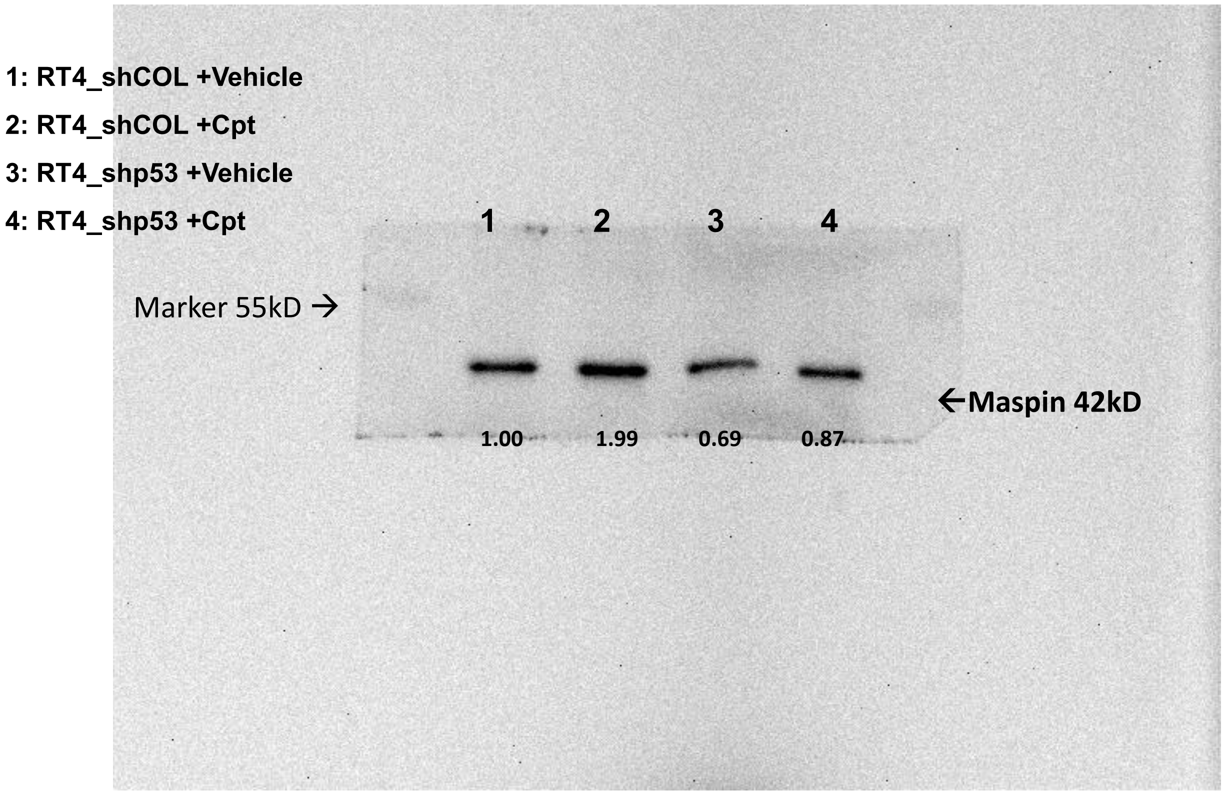

Figure supplementary 3. The original pictures of the immunoblot assays
