# Supplementary material for: Maspin is a PTEN-Upregulated and p53-Upregulated Tumor Suppressor Gene and Acts as an HDAC1 Inhibitor in Human Bladder Cancer
Source: Cancers (Basel). 2019 Dec 18;12(1):10. doi: 10.3390/cancers12010010 (PMC7016534; doi:10.3390/cancers12010010)
Supplement: Supplementary file 1 [file cancers-12-00010-s001.zip › Table S1.pdf]

Table supplementary 1. Total HDAC1 activity (pmol/min/mg protein)

	RT4_shCOL		RT4-shMaspin	
**n=3**	**IgG**	**HDAC1**	**IgG**	**HDAC1**
**1**	171.76	396.18	98.11	393.94
**2**	174.82	404.48	99.08	401.13
**3**	177.32	413.92	100.39	409.97

	T24-DNA		T24-Maspin	
**n=3**	**IgG**	**HDAC1**	**IgG**	**HDAC1**
**1**	193.48	510.99	160.09	371.80
**2**	195.45	518.51	161.33	379.52
**3**	199.15	530.81	164.52	388.01
